# Diagnosing the Dynamics of Observed and Simulated Ecosystem Gross Primary Productivity with Time Causal Information Theory Quantifiers

**DOI:** 10.1371/journal.pone.0164960

**Published:** 2016-10-20

**Authors:** Sebastian Sippel, Holger Lange, Miguel D. Mahecha, Michael Hauhs, Paul Bodesheim, Thomas Kaminski, Fabian Gans, Osvaldo A. Rosso

**Affiliations:** 1 Max Planck Institute for Biogeochemistry, Jena, Germany; 2 Norwegian Institute of Bioeconomy Research, Ås, Norway; 3 Instituto de Física, Universidade Federal de Alagoas, Maceió, Alagoas, Brazil; 4 German Centre for Integrative Biodiversity Research (iDiv), Leipzig, Germany; 5 Michael Stifel Center Jena for Data-Driven and Simulation Science, Jena, Germany; 6 University of Bayreuth, Bayreuth, Germany; 7 The Inversion Lab, Hamburg, Germany; 8 Instituto Tecnológico de Buenos Aires (ITBA) and CONICET, Ciudad Autónoma de Buenos Aires, Argentina; 9 Complex Systems Group, Facultad de Ingeniería y Ciencias Aplicadas, Universidad de los Andes, Las Condes, Santiago, Chile; Pacific Northwest National Laboratory, UNITED STATES

## Abstract

Data analysis and model-data comparisons in the environmental sciences require diagnostic measures that quantify time series dynamics and structure, and are robust to noise in observational data. This paper investigates the temporal dynamics of environmental time series using measures quantifying their information content and complexity. The measures are used to classify natural processes on one hand, and to compare models with observations on the other. The present analysis focuses on the global carbon cycle as an area of research in which model-data integration and comparisons are key to improving our understanding of natural phenomena. We investigate the dynamics of observed and simulated time series of Gross Primary Productivity (GPP), a key variable in terrestrial ecosystems that quantifies ecosystem carbon uptake. However, the dynamics, patterns and magnitudes of GPP time series, both observed and simulated, vary substantially on different temporal and spatial scales. We demonstrate here that information content and complexity, or Information Theory Quantifiers (ITQ) for short, serve as robust and efficient data-analytical and model benchmarking tools for evaluating the temporal structure and dynamical properties of simulated or observed time series at various spatial scales. At continental scale, we compare GPP time series simulated with two models and an observations-based product. This analysis reveals qualitative differences between model evaluation based on ITQ compared to traditional model performance metrics, indicating that good model performance in terms of absolute or relative error does not imply that the dynamics of the observations is captured well. Furthermore, we show, using an ensemble of site-scale measurements obtained from the FLUXNET archive in the Mediterranean, that model-data or model-model mismatches as indicated by ITQ can be attributed to and interpreted as differences in the temporal structure of the respective ecological time series. At global scale, our understanding of C fluxes relies on the use of consistently applied land models. Here, we use ITQ to evaluate *model structure*: The measures are largely insensitive to climatic scenarios, land use and atmospheric gas concentrations used to drive them, but clearly separate the structure of 13 different land models taken from the CMIP5 archive and an observations-based product. In conclusion, diagnostic measures of this kind provide data-analytical tools that distinguish different types of natural processes based solely on their dynamics, and are thus highly suitable for environmental science applications such as model *structural* diagnostics.

## Introduction

Understanding the feedback between terrestrial ecosystems and changing environmental conditions is a key prerequisite to model the impact of global change. Of particular relevance are changes in the global carbon (C from now on) cycle, i.e. in land-atmosphere fluxes and thus biosphere carbon stocks (e.g. [1]). Today, empirical and process-based models of varying structural and numerical complexity are used to assess and predict past, present and future ecosystem-atmosphere carbon exchange (e.g. [2]).

The state of current process-based understanding is in part implemented in terrestrial biosphere models. The process representations in these models differ widely, which is in part due to insufficient mechanistic understanding of the underlying processes or uncertain parameter estimates. This lack of consensus leads to major uncertainties in the predictions [3]. The starting point to reduce uncertainties in modelled fluxes or physiological state variables is to quantify their mismatch with observations [4].

Model *benchmarking* seeks to quantify this mismatch and to rank the models accordingly [5–9]. Obviously, model rankings depend on the metric(s) considered; it is unlikely that one single model will rank best with respect to multiple metrics that may emphasize different aspects.

Activities such as the International Land Model Benchmarking Project (ILAMB, http://ilamb.org) have formalized this idea by providing a range of benchmarks. In order to standardize the benchmarking approaches further, several platforms for automatic benchmarking have been developed [2, 7]. Most of the metrics used for benchmarking so far are straightforward, for instance focusing on long-term mean values per variable and pixel (or region), or the amplitude of some specific seasonal cycle (e.g. of some land-atmosphere flux). However, metrics of this kind can only provide a limited insight into the dynamics of the models under scrutiny. Consequently, latest benchmarking efforts intend to consider relevant observed and simulated patterns, such as process efficiencies or turnover-rates [10], i.e. these approaches attempt to derive a pattern-oriented strategy to model evaluation and model-data integration [11–13]. Hence, various initiatives to land surface model benchmarking are still working towards developing a widely acceptable set of benchmarks [8] that target various relevant aspects of model behaviour.

One difficulty in model benchmarking is that reference data are potentially affected by observational noise or biases. Ideally, models should exhibit similar dynamics as the observations on a range of time scales [14]. Disagreements of models and observations indicate either inaccurate parameter estimates, structural deficits, or other inadequacies of the models; or they originate in low quality of the reference observations, with e.g. noise contamination at high frequencies and sensor ageing processes at long time scales. Hence, in any model-data comparison such as benchmarking, a distinction between signal and noise is necessary. In the interpretation of data analysis, signal extraction from the noise background is key; process-oriented models should be able to reproduce the signal but not the noise part of observations.

Here, we contribute to the discussion of model evaluation and benchmarking by investigating the potential of *Information Theory Quantifiers* (ITQ) as an additional set of benchmarking tools, with a special focus on the dynamics and structure of model simulated vs. observed time series. For example, questions such as *‘How much information do the observations reveal about the dynamics of the underlying system or processes? Do observed time series resemble stochastic or deterministic processes? Are models reproducing the observed process classes?’* arise naturally from an Information Theory perspective and could potentially be tackled using ITQ. These measures draw a distinction between deterministic and stochastic processes, are complementary to the present set of tools and might thus provide a balanced investigation of model evaluation, e.g. sensu [6].

Among the set of variables established to describe C dynamics, the gross CO_2_ uptake of terrestrial vegetation from the atmosphere (‘Gross Primary Productivity’, or GPP) is of particular importance for terrestrial ecosystems. GPP can be derived from in-situ measurements of net ecosystem exchange fluxes of CO_2_. GPP is predominantly controlled by radiation, but likewise sensitive to temperature, CO_2_ concentrations, water availability and the phenological status of the vegetation, amongst other factors [15]. GPP is routinely obtained as the difference of Net Ecosystem Productivity (NEP) measurements and estimates for ecosystem respiration (*R*_*eco*_) at individual sites as part of a global monitoring network [16]. GPP is expected to show seasonal variation; abrupt changes or systematic trends in GPP can be induced by land use change, extreme events, and changing climate, among other factors. Numerous attempts to quantify GPP at larger spatial scales exist, but many of these rely on dynamical global vegetation and biogeochemical models. Based on assumed biological and physical processes, the latter either try to reconstruct historical GPP time series, based on observed precipitation, radiation, temperature and other drivers, or project the future dynamics of GPP using climate scenarios (Representative Concentration Pathways, RCPs [17]). Generally, GPP simulations respond globally to transient climate change, but locally and regionally also to abrupt land use changes, or extreme hydrometeorological anomalies [18, 19].

This study aims to introduce and interpret ITQ in the context of environmental time series analysis with particular emphasis on model evaluation and benchmarking of GPP dynamics at site, continental and global scale. The paper is organized as follows: the Section Quantifiers from Information Theory” provides an introduction to the field of ordinal pattern statistics and the associated Information Theory Quantifiers. “Data Sets Investigated” introduces the data: observations and model simulations of GPP at the site, continental and global scale; and a remotely sensed widely used proxy for vegetation activity (‘Fraction of Absorbed Photosynthetically Active Radiation’ (FAPAR)). The subsection “An Information Theory perspective on spatio-temporal environmental datasets” provides an intuitive introduction to ITQ in the context of environmental science using i) a FAPAR dataset at continental-scale and high spatial resolution; and ii) in-situ GPP measurements from flux tower sites to illustrate the effects of temporal resolution from the FLUXNET database, cf. http://fluxnet.fluxdata.org [16]. Then, we proceed by comparing the complexity and information content of GPP time series simulated by two process-based ecosystem models and an observations-based dataset at continental scale in a spatially explicit manner; these results are augmented with site-scale flux tower measurements. In “Global analysis of land surface models”, ITQ calculated from global-scale model simulations of GPP obtained from a climate model intercomparison project (CMIP5) comprising both historical reconstructions and climate scenario runs (1861-2090; ‘scenarios’) are evaluated to diagnose model structure. Finally, we conclude on the suitability of Information Theory Quantifiers for environmental science applications, such as diagnosing model structure or model benchmarking.

## Quantifiers from Information Theory

Given a time series or other observational data, a natural question arises: how much information are these data revealing about the dynamics of the underlying system or processes? The information content of data sets is typically evaluated via characterizing a value distribution or a probability density function (PDF) *P* describing the apportionment of some measurable or observable quantity [20]. These quantifiers represent metrics on the space of PDFs for data sets, allowing to compare different sets and to classify them according to the properties of underlying processes—broadly, stochastic vs. deterministic. Here, we refer to stochastic processes like correlated noise on one hand, and to deterministic chaotic maps which do not contain any noise on the other. Time series generated by these two categories of processes are difficult to discern by conventional analysis, but are clearly separated by ITQ [21].

In our case, we are interested in the temporal dynamics of GPP, and our data are *time series* *x*(*t*). Thus, we are mostly interested in metrics which take the temporal order of observations explicitly into account; i.e. the approach is fundamentally a *causal* rather than a *statistical* one. In a purely statistical approach, correlations between sucessive values from the time series are ignored or simply destroyed via construction of the PDF; while a causal approach focuses on the PDFs of data sequences.

The quantifiers selected are based on ordinal pattern statistics (cf. e.g. [22]). For an application of alternative quantifiers based on Symbolic Dynamics to environmental data, we refer to [23]. The quantifiers used here belong to either of two broad categories: those which quantify the *information content* of data versus those related to their *complexity* on one hand; and metrics related to *global* properties of the appropriate PDFs versus ones which take *local* properties into account. Note that we are referring to the space of probability density functions here, not physical space. For the sake of clarity and simplicity, we introduce Information Theory quantifiers that are defined on discrete PDFs in this section, since we are only dealing with discrete data (time series). However, all the quantifiers also have definitions for the continuous case [24, 25].

### The Shannon entropy as a measure for information content

The permutation Shannon entropy is a measure for the information content of the time series [24] and quantifies uncertainty, disorder, state-space volume, and lack of information [26]. Let *P* = {*p*_*i*_; *i* = 1, …, *N*} with $\sum_{i=1}^{N}p_{i}=1$, be a probability distribution, with *N* possible states of the system under study. The Shannon information measure (entropy) reads

$$S\left[P\right]\;=\;-\sum_{i=1}^{N}p_{i}\ln[p_{i}]\;.$$ (1)

The Shannon entropy is minimal when all but one of the *p*_*i*_’s vanish, and maximal when all *p*_*i*_’s are equal, i.e. for the uniform distribution $P_{e}=\left\{p_{i}=\frac{1}{N},\forall i=1,\ldots ,N\right\}$. In this case, $S\left[P_{e}\right]=S_{\max}=\ln N$. However, these two situations are extreme cases, unlikely to occur in any natural phenomenon considered here. In the following, we focus on the ‘normalized’ Shannon entropy, 0≤H≤1, given as

$$H\left[P\right]\;=\;S\left[P\right]/S_{\max}\;.$$ (2)

### Statistical complexity measures

Contrary to information content, there is no universally accepted definition of complexity. Here, we focus on describing the *complexity of time series* and do not refer to the complexity of the underlying *systems*. In fact, “simple” models might generate complex data, while “complicated” systems might produce output data of low complexity [27].

**Fig 1:**
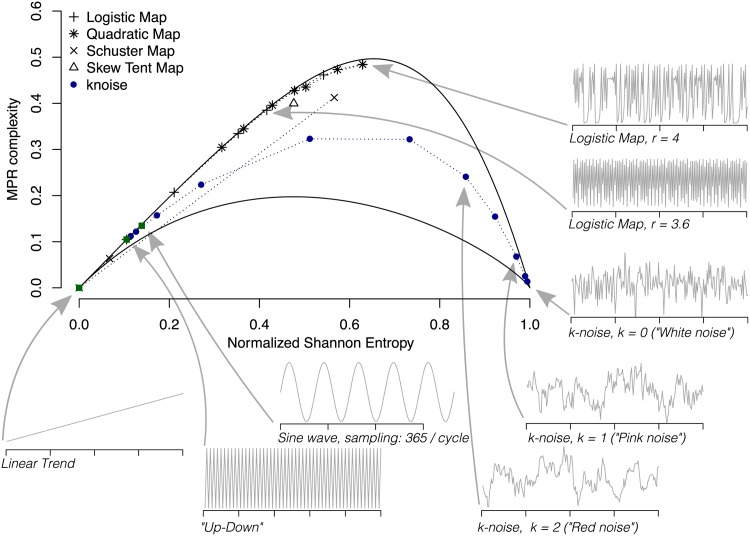
Illustration of causal Information Theory quantifiers as data-analytical tools. The *causality entropy–complexity plane* exhibits upper and lower limit curves, and the distances to these can be used to classify relevant processes. For all calculations, *D* = 6, *τ* = 1, and *n* = 10^4^ were chosen.

An intuitive notion of a quantitative complexity attributes low values both to perfectly ordered data (i.e. with vanishing Shannon entropy) as well as to uncorrelated random data (with maximal Shannon entropy), as both cases can be well described in a compact manner. For example, the statistical complexity of a simple oscillation or trend (ordered), but also of uncorrelated white noise (disordered) would be classified as low. Hence, linear transient trends and measurement noise of some geophysical variable would exhibit small complexity values, but the two processes would differ considerably in their Shannon entropy (Fig 1). Between the two cases of minimal and maximal entropy, data are more difficult to characterize and hence the complexity should be higher. We seek some functional C[P] quantifying structures present in the data which deviate from these two cases. These structures relate to organization, correlational structure, memory, regularity, symmetry, patterns, and other properties [28].

One suitable way to guarantee the desired properties for a complexity measure is to build the product of a measure of information and a measure of disequilibrium, i.e. some kind of distance from the uniform (‘equilibrium’) distribution of the accessible states of a system. In this respect, in [29] an effective *Statistical Complexity Measure* (SCM) C was introduced, that is able to detect and discern basic dynamical properties of datasets.

Based on the seminal notion advanced by Lopez-Ruiz *et al.* [30], this statistical complexity measure [29, 31] is defined through the functional product form

$$C\left[P\right]\;=\;Q_{J}\left[P,P_{e}\right]\cdot H\left[P\right]$$ (3)

of the normalized Shannon entropy H, see Eq (2), and the disequilibrium $Q_{J}\left[P,P_{e}\right]$ defined in terms of the Jensen-Shannon divergence $J[P,P_{e}]$. The latter is given by

$$Q_{J}\left[P,P_{e}\right]\;=\;Q_{0}J\left[P,P_{e}\right]\;=\;Q_{0}\left\{S\left[\left(P+P_{e}\right)/2\right]-S\left[P\right]/2-S\left[P_{e}\right]/2\right\},$$ (4)

where *Q*_0_ is a normalization constant chosen such that $0\leq Q_{J}\leq 1$:

$$Q_{0}\;=\;-2{\left\{\frac{N+1}{N}\ln\left(N+1\right)-\ln\left(2N\right)+\ln N\right\}}^{-1}\;.$$ (5)

The inverse of this value is obtained for the non-normalized Jensen-Shannon divergence when one of the components of *P*, say *p*_*m*_, is equal to one and the remaining *p*_*j*_’s are zero.

The Jensen-Shannon divergence quantifies the difference between probability distributions and is especially useful to compare the symbolic composition of different sequences [32]. Note that the above introduced Information Theory quantifier depends on two different probability distributions: one associated with the system under analysis, *P*, and the other the uniform distribution *P*_*e*_. For the latter, other reference distributions can be chosen to test whether an observed distribution is close to a target distribution. It has been shown that there are *limit curves* for complexity: for a given value of H and any data set, the possible C values vary between a minimum $C_{min}\left(H\right)$ and a maximum $C_{max}\left(H\right)$, restricting the possible values of the complexity measure [33].

An alternative measure which is local in distribution space is the Fisher Information Measure (FIM). For calculating FIM, we use probability amplitudes as a starting point [25] and obtain a discrete normalized FIM, 0≤F≤1, convenient for our purposes [34]:

$$F\left[P\right]\;=\;F_{0}\sum_{i=1}^{N-1}{\left[\sqrt{p_{i+1}}-\sqrt{p_{i}}\right]}^{2}\;.$$ (6)

Here the normalization constant *F*_0_ reads

$$F_{0}\;=\;\{\begin{array}{cc} 1, & \text{if}\;p_{i^{*}}=1\;\text{for}\;i^{*}=1\;\text{or}\;i^{*}=N\;\text{and}\;p_{i}=0,\forall i\neq i^{*},\\ 1/2, & \text{otherwise}\;. \end{array}$$ (7)

The general behavior of the this discrete version of the FIM (Eq (6)) is opposite to that of the Shannon entropy, except for periodic motions. The local sensitivity of FIM for discrete PDFs is reflected in the fact that it depends on the specific ‘*i*–ordering’ of the discrete values *p*_*i*_ [34, 35]. The summands in Eq (6) can be regarded as a ‘distance’ between two contiguous probabilities. Thus, a different ordering of the patterns would lead to a different FIM-value, demonstrating its local nature. In the present work, we follow the so-called Keller sorting scheme [36] for the generation of the Bandt and Pompe PDF discussed in the next section.

### The Bandt and Pompe approach to generate a causal PDF

The quantifiers from Information Theory rely on a probability distribution associated to the time series. The determination of the most adequate PDF is a fundamental problem because the PDF *P* and its sample space *Ω* are inextricably linked. The usual histogram technique is inadequate since the data are treated purely stochastic and the temporal information is completely lost. Bandt and Pompe (BP) [22] introduced a simple and robust symbolic methodology that takes into account time ordering of the time series by comparing neighboring values in a time series. The symbolic data are created by first ranking the values of the series within windows of a fixed length, and then reordering these embedded data in ascending order. This is tantamount to a phase space reconstruction with embedding dimension (pattern length) *D* and time lag *τ* (see Text 1 in S1 File for a more detailed description). In this way, it is possible to quantify the diversity of the ordering symbols (patterns) derived from a scalar time series. Note that the appropriate symbol sequence arises naturally from the time series, and no model-based assumptions are needed. As such, it allows to uncover important details concerning the ordinal structure of the time series [21] and can also yield information about temporal correlation [37].

This type of analysis of a time series entails losing details of the original series’ amplitude information. However, the symbolic representation of time series by recourse to a comparison of consecutive (*τ* = 1) or nonconsecutive (*τ* > 1) values allows for an accurate empirical reconstruction of the underlying phase-space, even in the presence of weak (observational and dynamic) noise [22]. Furthermore, the ordinal patterns associated with the PDF are invariant with respect to nonlinear monotonous transformations; nonlinear drifts or scaling artificially introduced by a measurement device will not modify the estimation of quantifiers, a nice property if one deals with experimental data (see, e.g., [38]). The only condition for the applicability of the BP method is a very weak stationarity assumption: for *k* ≤ *D*, the probability for *x*_*t*_ < *x*_*t*+*k*_ should not depend on *t*. For a review of the BP methodology and its applications to physics, biomedical and econophysics signals, see [39].

**Fig 2:**
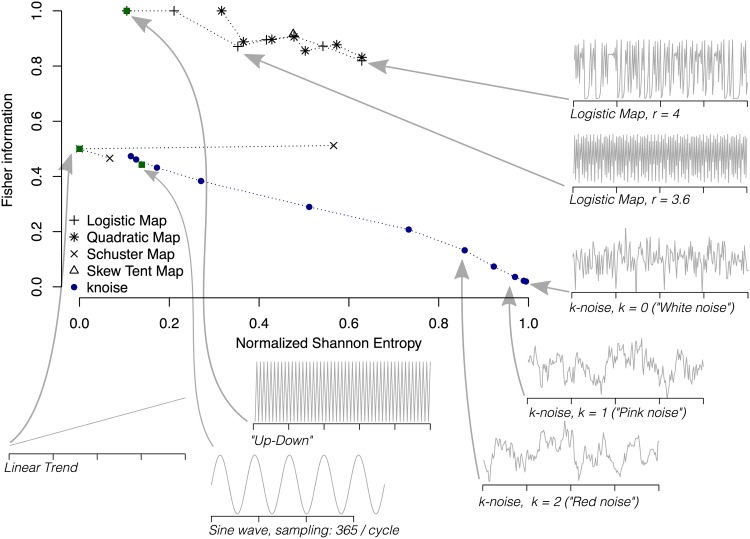
Illustration of the *causality Shannon-Fisher plane* plane (H×F) as data-analytical tool. H×F is used to classify relevant processes, such as constant/periodic signals, white and coloured noise (noise with a power spectrum proportional to 1/*f*^*k*^), and deterministic signals (e.g. chaotic maps), similar to Fig 1. For all calculations, *D* = 6, *τ* = 1, and *n* = 10^4^ were chosen.

Regarding the selection of the parameters, Bandt and Pompe suggested working with 4 ≤ *D* ≤ 6 for typical time series lengths, and specifically considered a time lag *τ* = 1 in their cornerstone paper [22]. For the artificially generated time series shown below (Figs 1 and 2), we chose *D* = 6 and follow the Lehmer-permutation scheme [35] to calculate the Fisher Information. For the measured and modelled time series analyzed here, the embedding dimension is chosen as *D* = 4 throughout due to time series length requirements (S1 File), in particular at coarse temporal resolution, and to achieve comparability across the different analyses. In all cases, the delay parameter has been set to *τ* = 1.

### Incorporating amplitude information: Weighted ordinal pattern distribution

Recently, the permutation entropy was extended to incorporate also amplitude information [40]. Hence, a potential disadvantage of ordinal pattern statistics, namely the loss of amplitude information, can be addressed by introducing weights in order to obtain a ‘weighted permutation entropy (WPE)’. In the context of environmental time series, which typically exhibit a pronounced seasonal cycle and thus seasonally varying signal to noise ratios, this idea might be particularly useful to address (noisy) low-variance patterns (e.g. during dormancy periods in winter).

Weighting the probabilities of individual patterns according to their variance alleviates potential issues regarding to ‘high noise, low signal’ patterns, because low-variance patterns that are stronlgy affected by noise are down-weighted in the resulting ‘weighted ordinal pattern distributions’. For example, [40] show that a weighted entropy measure is sensitive to sudden changes in the variance of the time series. Here, we extend the idea of WPE following [40] to derive a weighted permutation entropy ($H_{w}$), weighted statistical complexity ($C_{w}$), and weighted Fisher Information ($F_{w}$).

Non-normalized weights are computed for each temporal window for the time series *X*, such that

$$w_{j}=\frac{1}{D}\sum_{k=1}^{D}{\left(x_{j+k-1}-\bar{X_{j}^{D}}\right)}^{2}.$$ (8)

Here, the embedding dimension is denoted by *D* and $\bar{X_{j}^{D}}$ denotes the arithmetic mean of the time series in the current window with index *j*. Thus, the weight of each window of length D is given by its variance in the equation above. The weights *w*_*j*_ are then used to modify the relative frequencies of each ordinal pattern $\pi_{j}\left(X_{j}^{D}\right)$, with *N* − *D* + 1 windows, given by $X_{j}^{D}=\left(x_{j},x_{j+1},...,x_{j+D-1}\right)$, for a time series of length *N* [40]:

$$p_{w}\left(\pi_{i}\right)=\frac{\sum_{j=1}^{N-D+1}\delta_{\pi_{i},\pi_{j}}w_{j}}{\sum_{k=1}^{N-D+1}w_{k}}$$ (9)

The denominator of this equation provides the normalization, and the Kronecker delta *δ*_*π*_*i*_*π*_*j*__ in the enumerator serves to indicate which pattern occurs in each window *j*:

$$\begin{array}{c} \delta_{\pi_{i},\pi_{j}}=\{\begin{array}{cc} 1, & \text{if}\;\pi_{i}=\pi_{j},\\ 0, & \text{if}\;\pi_{i}\neq \pi_{j}. \end{array} \end{array}$$

After having calculated the appropriate *π*_*i*_ for each *i* = 1, …, *D*!, the same Eqs (2), (3) and (6) are applied to obtain the weighted versions $H_{w}$, $C_{w}$ and $F_{w}$ of the ITQ.

The weighting of the ITQ can be considered as noise filter, provided that noise is characterized by relatively low variance, and thus enhances the signal contained in the time series. In any case, rare patterns are suppressed in favour of more frequent ones.

Obviously, the window-based variance is but one out of many weighting recipes, others are easily conceivable. There is also a connection to the celebrated Rènyi entropy ([41])

$$\begin{array}{c} H_{q}\left(X\right)=\frac{1}{1-q}\log_{2}\left(\sum_{i=1}^{D!}p_{i}^{q}\right) \end{array}$$

where the Rènyi exponent *q* suppresses low-frequency patterns for *q* > 1; for *q* = 1, the Shannon entropy is obtained. The resulting ‘Rènyi ordinal pattern distribution’ could be considered as a special case of the weighted pattern distribution, characterized by a single exponent; this has not been investigated so far, however.

Summarizing, ITQ computed based on an appropriately weighted form of the ordinal pattern distribution are suitable to analyse data sets with considerable amplitude information (e.g. seasonal variation) from an information-theoretic viewpoint. Since this is an issue for the time series investigated here, we will mostly perform our ITQ analysis using the weighted versions described here.

### Causal information planes

A particularly useful visualization of the quantifiers from Information Theory is their juxtaposition in two-dimensional graphs (‘*causal information planes*’), e.g. *a)* The *causality entropy–complexity plane*, H×C, or its variance-weighted variant $H_{w}\times C_{w}$, is based only on global characteristics of the associated time series PDF (both quantities are defined in terms of Shannon entropies); while *b)* the *causality Shannon-Fisher plane*, H×F, or its variance-weighted variant ($H_{w}\times F_{w}$), is based on global and local characteristics of the PDF. In the case of H×C (or its weighted version) the value range is $\left[0,1\right]\times [C_{min}\left(H\right),C_{max}\left(H\right)]$, while in the causality plane H×F (or with weights) the range is presumably [0, 1] × [0, 1]; no limit curves have been shown to exist so far.

These diagnostic planes are particularly efficient to distinguish between the deterministic chaotic and stochastic nature of a time series [21, 34] since the permutation quantifiers have distinct behaviours for different types of processes, see Figs 1 and 2, respectively.

Chaotic maps have intermediate entropy H and Fisher F values, while their complexity C reaches larger values, very close to the upper complexity limit [21, 34]. For regular processes, entropy and complexity have small values, close to zero, while the Fisher information is close to one. Uncorrelated stochastic processes have H near one and C, F near zero, respectively. It has also been found that correlated stochastic noise processes with a power spectrum proportional to 1/*f*^*k*^, where 1 ≤ *k* ≤ 3, are characterized by intermediate permutation entropy and intermediate statistical complexity values [21], as well as intermediate to low Fisher information [34]. In both causal information planes (H×C, see Fig 1 and H×F, see Fig 2), stochastic data are clearly localized at different planar positions than deterministic chaotic ones. These two causal information planes have been profitably used to visualize and characterize different dynamical regimes when the system parameters vary [34, 35, 42–51]; to study temporal dynamic evolution [52–54]; to identify periodicities in natural time series [55]; to identify deterministic dynamics contaminated with noise [56, 57]; to estimate intrinsic time scales and delayed systems [58–60]; for the characterization of pseudo-random number generators [61, 62]; to quantify the complexity of two-dimensional patterns [63]; and for ecological [45], biomedical and econophysics applications (see [39] and references therein).

### Quantification of distance between models and observations

The overall objective of this paper is to provide a methodology for analyzing time series and comparing models with data based on Information Theory Quantifiers. However, the quantification of visually observed differences in causal information planes is not completely straightforward. Since the information planes are not Euclidean spaces, the Euclidean distance between pairs of points is not suitable. We need a distance metric that takes the nonlinear structure of the manifold into account. As we are working in the space of ordinal pattern distributions, a distance measure between PDF’s is appropriate. We quantify the discrepancy between observations and the model outputs by calculating the Jensen-Shannon divergence [64] between them:

$$\begin{array}{c} J\left[P_{obs},P_{mod}\right]=S\left[\left(P_{obs}+P_{mod}\right)/2\right]-S\left[P_{obs}\right]/2-S\left[P_{mod}\right]/2, \end{array}$$ (10)

where *P*_*obs*_ and *P*_*mod*_ are the ordinal pattern distributions of the observations (or ‘observation-based products’, see next section) and the model outputs, respectively.

We note that a model evaluation based on Information Theory Quantifiers directly on the model residuals (*X*_*t*,*Mod*_ − *X*_*t*,*Obs*_) is not meaningful in itself: the ordinal pattern distribution of the model residuals for a poor, but noisy model with high variance would exhibit low complexity and high entropy values (‘white noise’)—despite an inadequate model structure in this simple example. That model residuals exhibit white noise behaviour is not an indicator for a good model performance per se.

### Data analysis and software

The open source R-package “statcomp” (Statistical Complexity Analysis) has been written to facilitate an easy access to ITQ’s and is available on CRAN (http://CRAN.R-project.org/package=statcomp) and R-Forge (http://r-forge.r-project.org/projects/statcomp/). A short installation guide, links to a detailed manual and a code tutorial to reproduce Figs 1 and 2 are given in S1 Code.

## Data Sets Investigated

### A remotely sensed vegetation activity proxy: FAPAR

The ‘Fraction of Absorbed Photosynthetically Active Radiation’ (FAPAR) is a variable that describes the ratio of absorbed to total incoming solar radiation in the photosynthetically active wavelength range at the land surface [65]. As the solar radiation is the driver for photosynthetic activity, FAPAR is used to diagnose vegetation productivity (e.g. [66]) and a so-called essential climate variable for global monitoring of the land surface and the terrestrial biosphere [67]. Here we use the JRC-TIP FAPAR data set [65] to provide an intuitive introduction and interpretation of continental-scale gradients and structure as obtained from analyses using ITQ (cf. above). This FAPAR product is derived (together with a set of further land surface variables such as the effective Leaf Area Index and the albedo of the soil background) by the Joint Research Centre Two-Stream Inversion Package (JRC-TIP), which is based on a one-dimensional (Two-Stream) representation of the canopy-soil system. The products were retrieved in an inversion procedure that combines the information in the MODIS broadband albedo product and prior information on the model’s state variables. For the purpose of our analysis it is worth noting that the prior information is constant in space and time with the exception of snow events, i.e. the spatio-temporal structure in the FAPAR data set is solely imposed by observations from space. The use of the two-stream model ensures physical consistency of all derived variables, as long as the products are used in the native resolution of the albedo input product [68], which in our case is 1km. In the temporal domain, as for the output of the terrestrial model (see below) we use monthly averages.

### GPP time series at site-scale: Flux tower measurements

Fluxes across the atmosphere-biosphere boundary (directly above the canopy) are measured routinely in a global network of flux tower sites (FLUXNET) using the Eddy-Covariance (EC) method [16]. Net ecosystem fluxes of carbon were partitioned into GPP and ecosystem respiration by using nighttime data that consist only of respiratory fluxes [69].

In this study, the dynamics of GPP time series from an ensemble of European FLUXNET sites with each more than five years of continuous measurements are investigated at monthly resolution. In addition, three sites are selected to illustrate the effect of temporal resolution (i.e. aggregation from half-hourly to monthly resolution) on complexity and entropy of the respective time series. These three sites represent different major European climate regions, i.e. Mediterranean evergreen broadleaf (Puechabon, France), temperate and boreal evergreen needleleaf (Tharandt, Germany and Hyytiälä, Finland, respectively) forest sites. A table containing detailed information about all investigated sites is available (Table 1 in S1 File).

### Continental-scale estimates of GPP: comparison of model runs and an observations-based product

#### Model Tree Ensembles (MTE)

An empirical upscaling of GPP fluxes from the site scale to global scales was conducted by [70]. These authors used Fluxnet site measurements with local meteorological observations and remotely sensed vegetation indices to train an ensemble of model trees. In a subsequent step, the model trees were used to predict spatially explicit, fully data-driven GPP fluxes (at 0.5° spatial and monthly temporal resolution, 1982-2011) using global, gridded meteorological data and remote sensing observations. These interpolated and upscaled GPP fluxes comprise the ‘observations’, as opposed to the model runs described in the following paragraph which are ‘simulations’. Two variants of the MTE dataset are used in this study, which differ in the method applied for partitioning the tower-based net flux measurements (Net Primary Productivity, or NPP) into GPP and ecosystem respiration, R_*eco*_. These include the widely used extrapolation of night-time respiration into daytime [69] (‘MTE-MR’), and a separation method that uses a light response curve [71] (‘MTE-GL’).

#### LPJml

The Lund-Potsdam-Jena managed Land dynamic global vegetation model (LPJmL) simulates dynamic vegetation development and structure of 10 natural plant functional types, two of which are herbaceous and eight are woody [72]. The human land use scheme consists of 13 crop functional types, including both grazing lands and agricultural crops [73]. Photosynthetic carbon assimilation in LPJmL follows the process-oriented coupled photosynthesis and water balance scheme of the BIOME3 model [74]. Photosynthesis is simulated at the canopy scale depending on seasonally varying nitrogen content and carboxylation capacity, which are functions of absorbed photosynthetically active radiation, temperature, atmospheric CO_2_, day length, and canopy conductance (ibid.).

#### JSBACH

The Jena Scheme for Biosphere-Atmosphere Coupling in Hamburg (JSBACH) is a modular land surface scheme based on the ‘Biosphere Energy-Transfer and Hydrology Model’ (BETHY, [75]). It comprises 13 natural plant functional types that are distinguished by plant (eco-) physiological properties, and the relevant spatial characteristics are prescribed as maps [76]. The model operates internally with 30 minutes temporal resolution. Model grid cells are covered by at most four different plant functional types [77]. Additionally, five vegetation phenotypes are specified, namely managed (non-forest) lands, grassland, raingreen forest or shrubland, evergreen and summergreen. Photosynthesis is simulated using distinct physiologically based submodules for C3 and C4 plants [78], and includes an explicit representation of the interdependence between the carbon assimilation rate and stomatal conductance [76]. Both variables are a function of temperature, soil moisture, water vapour, CO_2_ concentration and the absorption of visible solar radiation, the latter of which is resolved in three canopy layers.

#### Modelling Protocol

Both vegetation model simulations (LPJmL and JSBACH) were conducted at 0.25° spatial resolution and at the daily time scale for Europe [79]. Subsequently, the output was linearly aggregated to the monthly time scale and 0.5° spatial resolution for comparability with the MTE dataset. It is important to note that aggregation (taking mean values) and decimation (thinning to the desired resolution) of the time series are operations with very different consequences for the complexity analysis: whereas aggregation increases correlation, decreases Shannon entropy and in general induces a shift in the entropy-complexity plane to the left and upwards, decimation diminishes correlation, increases Shannon entropy and leads to a shift in the opposite direction.

### Global-scale simulated GPP dynamics: CMIP5

In order to evaluate the influence of different model structures vs. different climatic scenarios and trends on GPP dynamics, the behaviour of global GPP dynamics as simulated in the Fifth Climate Model Intercomparison Project (CMIP5) multimodel ensemble [3] is analyzed. The two representative concentration pathways (RCPs) 4.5 and 8.5 [17] are used in 13 different models and model variants (i.e. ensemble members) in monthly resolution from 1860-2099 (see Table 2 in S1 File for a detailed overview).

ITQ are calculated for each land grid cell in each of 59 model simulations (comprising combinations of model variants, different emission scenarios and ensemble members) and for each of the twenty-one 30-year periods within 1860-2099, yielding in total 1239 simulated 30-year periods. In addition, the two MTE datasets for the period 1982-2011 are included in the comparison. Subsequently, for each 30-year period, all grid cells are visualized in the causal information planes (see S6 and S7 Figs for an example), where each model-scenario combination generates a point cloud in the causal information plane.

To compare these point clouds in a rigorous manner, i.e. to take the different local point densities into account, the planes are rasterized using a regular grid of 25x25 pixels. Subsequently, the number of land grid cells that fall into each of the respective (up to 625) ‘causal information classes’ are counted and then normalized (to account for different spatial resolution), yielding one count ‘vector’ for each model-scenario combination. Note that due to the existence of the limit curves in H×C, some of the pixels are empty by necessity. All pixels with no points across all 1239 vectors are omitted, which yields an effective reduction in the vector’s dimensionality. Then, a principal component analysis (PCA) is conducted on these vectors that is used to illustrate in a simple manner the separation of CMIP5 models and scenarios along the first and second principal component. For H×F, we proceed in a similar manner.

## Results and Discussion

In this section, we first illustrate and interpret continental-scale gradients in ITQ as obtained from a high-resolution vegetation productivity proxy (FAPAR) and proceed by investigating the effect of temporal resolution on ITQ at site scale. Subsequently, we compare two models and an observations-based product, including an interpretation that might offer hints for model improvements. Finally, a global-scale application of complexity measures based on CMIP5 model simulations is presented.

### An Information Theory perspective on spatio-temporal environmental datasets

#### Continental-scale gradients in Information Theory Quantifiers

**Fig 3:**
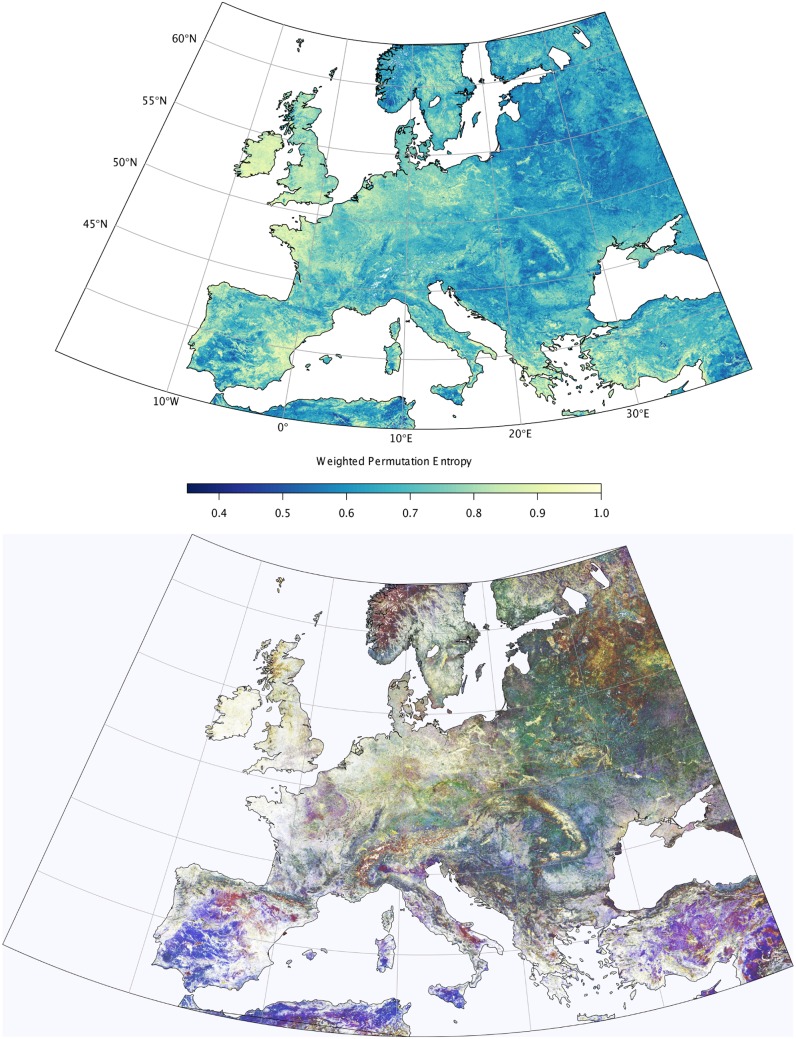
ITQ derived from monthly time series from the JRC-TIP FAPAR dataset. (top) Weighted Permutation Entropy, (bottom) RGB plot of weighted permutation entropy ($H_{w}$, red channel), weighted complexity ($C_{w}$, green channel), and weighted Fisher Information ($F_{w}$, blue channel). An intense color indicates a high value of the respective ITQ.

ITQ from time series of vegetation activity proxies such as FAPAR show a large variety of spatial patterns from local to continental scales due to differences in vegetation type, land use, climate, and other factors. For example, the Weighted Permutation Entropy ($H_{w}$) for monthly FAPAR time series over Europe shows large spatial gradients that are mainly related to the regularity of the seasonal cycle in vegetation activity (Fig 3, upper panel). Western parts of the continent and many coastal regions show rather high entropy values, e.g. over the British Isles, North-Western France, and the Netherlands, indicating a relatively stochastic behaviour of the respective time series. On the other hand, (North-)Eastern parts of the continent, but also seasonally dry regions like the Iberian peninsula, Southern Italy and North Africa exhibit low entropy values and thus monthly time series appear to be predictable. However, in Fig 3 it becomes already obvious that a quantification of entropy alone is not able to distinguish between climatologically and ecologically well-separated regions such as Southern Spain and Eastern Europe (both displaying low entropy).

Therefore, additional ITQ are needed and useful to distinguish further features in the time series, e.g. related to the separation of stochastic vs. deterministic processes [21]. Therefore, we exemplify the potential of combining several ITQ in the context of environmental time series in an RGB color image (Fig 3, bottom panel), where three ITQ (Weighted permutation entropy, statistical complexity, and Fisher Information) are encoded in the red, green, and blue channel, respectively (Fig 3, bottom panel). This picture (Fig 3, bottom panel, see S3 Fig for a high-resolution version, maps of individual ITQ are available in S1 and S2 Figs) illustrates that ITQ indeed capture a large amount of structure in environmental time series: Continental-scale gradients are clearly visible, but now structurally distinct regions (e.g. Southern Spain vs. Eastern Europe, see above) become well separated. Moreover, regional structures related to ecosystem type, vegetation seasonality, and topography (e.g. mountain ranges) appear. It should be noted that the patterns and gradients obtained result from dynamical behaviour, i.e. quantify properties of vegetation in time, whereas the relation to other aspects like water availability, soil type, climate zone, or topography is non trivial and they do not enter the calculation at all. This is a demonstration of the strength of the link between dynamical properties and environmental conditions for plant communities (the basis of plant biogeography). These results thus highlight the potential of ITQ as indicators of ecological structure, and for model-data comparison exercises (see next subsection).

#### The influence of seasonal and diurnal oscillations on statistical complexity

**Fig 4:**
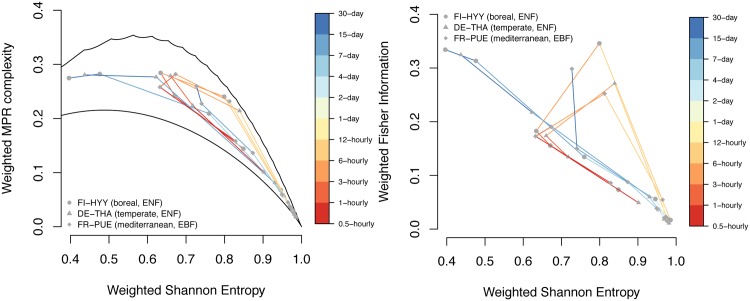
The effect of seasonal and diurnal oscillations on ITQ. GPP dynamics at three flux tower measurement sites from half-hourly to monthly resolution as quantified by a) $H_{w}\times C_{w}$, and b) $H_{w}\times F_{w}$.

Temporal resolution strongly affects the position of time series with oscillatory components in causal information planes. This property is illustrated for GPP time series from the boreal, temperate and Mediterranean climate regions (Fig 4). At the highest temporal resolution (half-hourly), GPP time series have a high $H_{w}$ and correspondingly small $C_{w}$ value, but this is distinctively different from the purely random case. At first, aggregating increases the complexity, until the strong daily cycle of the series is ‘detected’ at a resolution of 6 hours (due to *D* = 4, when one window covers just one day). The simple oscillatory behaviour reduces the complexity. The random extreme ($H_{w}$ close to one, $C_{w}$ close to zero) in the entropy-complexity plane is almost reached at daily resolution. Further aggregation increases $C_{w}$ and reduces $H_{w}$; at monthly resolution, we find an $H_{w}$ value around 0.65 only. We expect the classification in $H_{w}\times C_{w}$ to be most sensitive in this region in the center of the plane, since the difference between upper and lower limit curves is largest here. Thus, the monthly scale seems to be especially suitable for the subsequent regional assessment. It is not possible to further aggregate the series due to the length requirements—but at 3 months resolution, the annual cycle would be found, and another ‘loop’ in the diagram would be expected.

Similar to $H_{w}\times C_{w}$, the position of a time series in the Shannon-Fisher plane ($H_{w}\times F_{w}$) strongly depends on the temporal resolution (Fig 4). At the highest temporal resolution, the observed, EC-based GPP time series has a high $H_{w}≃0.95$ and a very small Fisher information. Aggregation decreases entropy and increases $F_{w}$ (Fig 4, upper part), until again the daily cycle of the series comes into reach at a resolution of 6 hours. Further aggregation increases entropy and decreases Fisher information, towards the “random corner” ($H_{w}$ close to one, $F_{w}$ close to zero) which is almost reached at daily and two-daily resolution. Aggregating further leads to a more and more steep increase in $F_{w}$, where the slope of this part of the curve resembles that of the *k*-noise shown in Fig 2.

### Model-data comparison using ITQ

**Fig 5:**
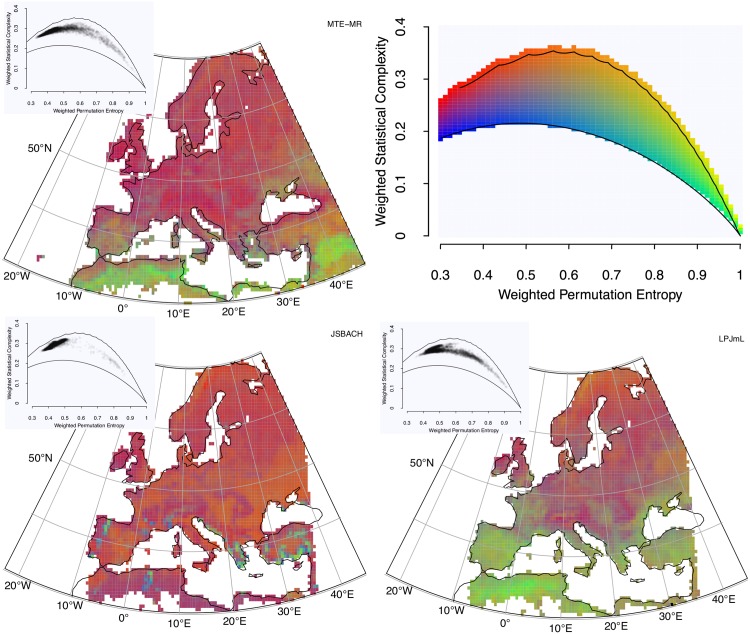
Model-Data comparison of simulated and observations-based GPP over the European continent. Colour-coded $H_{w}\times C_{w}$ (top right), as obtained from (top left) MTE-MR, (bottom left) JSBACH, and (bottom right) LPJmL. The causal information planes illustrating the point densities are given in the insets.

**Fig 6:**
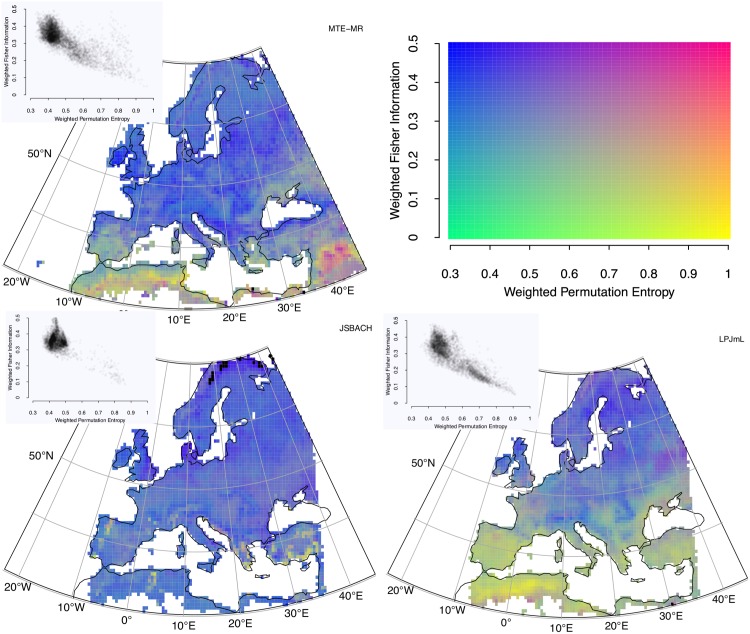
Model-Data comparison of simulated and observations-based GPP over the European continent. Colour-coded $H_{w}\times F_{w}$ (bottom right), as obtained from (top left) MTE-MR, (bottom left) JSBACH, and (bottom right) LPJmL. The causal information planes illustrating the point densities are given in the insets.

ITQ calculated from long-term GPP time series (1982-2011) at monthly resolution across Europe from two models and an observations-based product are shown in Fig 5 ($H_{w}\times C_{w}$ and Fig 6 $H_{w}\times F_{w}$), using a 2-dimensional colour scheme for visualization in a single map [80]. The observations-based product (MTE reconstructions, upper panels) indicate large, comparatively homogeneous regions across major biogeographical gradients, where the entropy of GPP time series is intermediate and their complexity medium to high. In the Mediterranean and in North Africa, the entropy is higher and the complexity and Fisher Information lower.

In the context of this comparison, MTE results are considered as “surrogate observations” or benchmarking datasets to be reproduced by the land surface models—a typical scenario in carbon cycle research [81, 82]. Accepting this assumption for the sake of comparison, models are considered “realistic” if their output is close to the MTE results (Figs 5 and 6). The first observation is that JSBACH produces a spatially very homogeneous ITQ field, whereas LPJmL exhibits a pronounced North-South gradient. A striking difference between the models is that JSBACH is more or less reproducing the ITQ from MTE, whereas LPJmL generates output which appears with higher information content than MTE. However, in seasonally dry regions such as southern parts of the Iberian Peninsula and North Africa, MTE and LPJmL seem to point in a similar direction towards higher entropy time series, whereas JSBACH does not show this feature. Thus, in these regions LPJmL performs better. These patterns likewise become obvious when looking at the density of points in the planes (insets), where JSBACH has much lower density in the high-entropy and low-complexity/low Fisher Information region than the observations; for LPJmL, the reverse holds true.

An interesting question in the context of model-data comparison using ITQ relates to whether the derived mismatch patterns (Figs 5 and 6) would be reproduced by traditional model performance metrics (e.g. the root mean squared error, RMSE).

**Fig 7:**
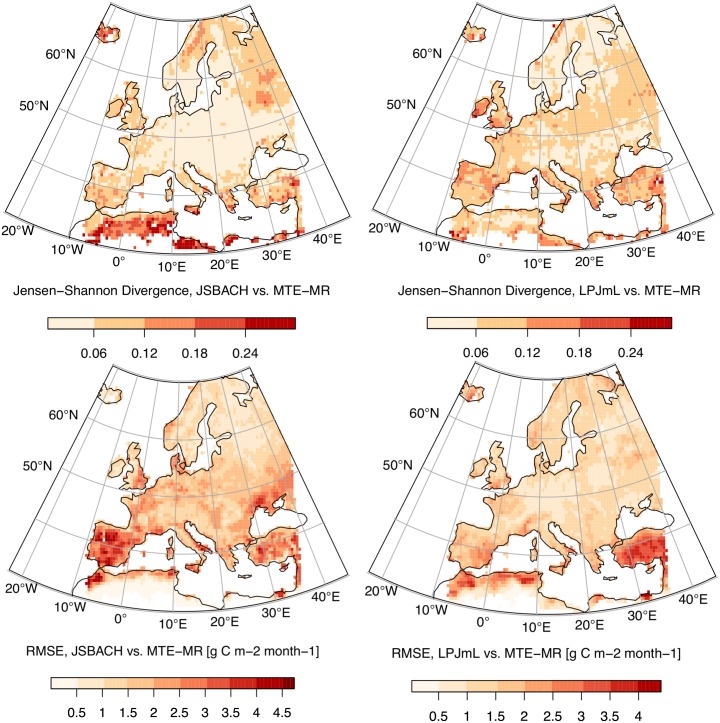
Model-Data comparison of simulated and observed GPP over the European continent. Jensen-Shannon Divergence between (A) JSBACH and MTE-MR, and (B) LPJmL and MTE-MR. RMSE between (C) JSBACH and MTE-MR, and (D) LPJmL and MTE-MR.

In Fig 7, colour-coded Jensen-Shannon divergences for the ordinal pattern distributions between the observation dataset MTE-MR and either of the two models are visualized and can be seen as an ITQ-based model performance metric. These maps demonstrate that JSBACH model output is rather close to the MTE-reconstructions in large parts of Central and Eastern Europe, but shows some deviations in Scandinavia, North-West Russia, and Northern Africa. In contrast, LPJmL is in better agreement with the MTE in Scandinavia, but LPJmL deviates from MTE in the Mediterranean and in several temperate European regions. Overall, LPJmL is the model with higher entropy values (at the monthly time scale) having a bias towards the random case (at least relative to MTE). JSBACH largely reproduces the behaviour of the MTE dataset, although it overemphasizes deterministic components in the Mediterranean and in the northern boreal.

While this analysis is useful to investigate deviations in the dynamics, as quantified by the ordinal pattern distributions, we emphasize that it can complement, but not replace traditional model evaluation metrics. This is because the ITQ analysis performed here cannot account for biases in the mean, variance or higher statistical moments of model output. A direct comparison between a conventional model evaluation metric (root mean squared error, RMSE) and the complexity-based metrics (i.e. Jensen-Shannon divergence) reveals that the latter indeed complements traditional model evaluation tools (Fig 7): For the majority of simulated grid cells, the two metrics behave opposite to each other, i.e. the model with a lower RMSE performs worse on the dynamics (i.e. higher Jensen-Shannon divergence), and vice versa (S4 Fig). Yet, the two metrics are not strongly anticorrelated (*R* = −0.296) and thus not redundant to each other.

Given the observed qualitative discrepancies between the “surrogate observations” and both models in their representation of GPP dynamics using ITQ, a crucial question arises: *Can these findings be explained or interpreted in an ecologically meaningful way?*

**Fig 8:**
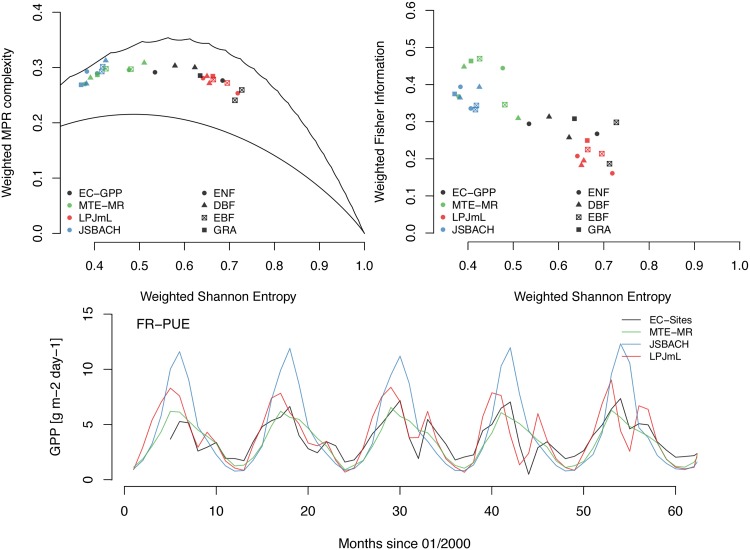
ITQ-based model-data comparison at site scale for Mediterranean flux tower sites. (A) $H_{w}\times C_{w}$, and (B) $H_{w}\times F_{w}$. (C) Time series of GPP at Puechabon, France.

To this end, we focus on the Mediterranean region because here the qualitative differences are most pronounced (Figs 5–7). Fig 8 shows an ensemble of Mediterranean flux tower observations (all sites with more than 5 years of continuous data, see Table 1 in S1 File), in addition to the respective pixels in MTE and the two models. The ensemble of sites in $H_{w}\times C_{w}$ and $H_{w}\times F_{w}$ (Fig 8) largely confirms the observed (spatial) pattern: In the Mediterranean, JSBACH simulations are very homogeneous, close to MTE, and these two point clouds cluster towards the more deterministic parts of the causal information planes. On the other hand, LPJmL tends to produce time series with higher information content, leading to a poor ITQ-based performance relative to MTE (see above). Surprisingly though, site-based GPP measurements tend to fall closer to LPJmL than to the other two datasets in the planes. This indicates that the observations-based MTE product (and JSBACH) do not necessarily match the site-level GPP measurements from an ITQ perspective. However, a ‘process-oriented’ interpretation of these ITQ-based patterns and discrepancies is feasible, considering, for example, the monthly GPP time series of an evergreen broadleaf forest site (Puechabon, France, Fig 8, bottom panel): Here, MTE and JSBACH indicate a very simple seasonal oscillation with only one (summer) peak per season. In contrast, flux tower measurements and LPJmL exhibit a two-peaked seasonal structure, with an early summer peak, subsequent GPP reduction due to water limitation, followed by a (smaller) autumn peak once water limitation ceases. Hence, in the model-data comparison presented above, ITQ readily diagnose these two contrasting dynamics, which can thus be used as a starting point to improve or optimize models and observations-based datasets.

In summary, these results show that model evaluation and improvement based solely on absolute or relative error metrics could be misleading if the dynamics are misrepresented in the model; in contrast a joint consideration of a variety of benchmarking metrics might provide useful hints to scrutinizing various aspects of model behaviour.

### Global analysis of land surface models

**Fig 9:**
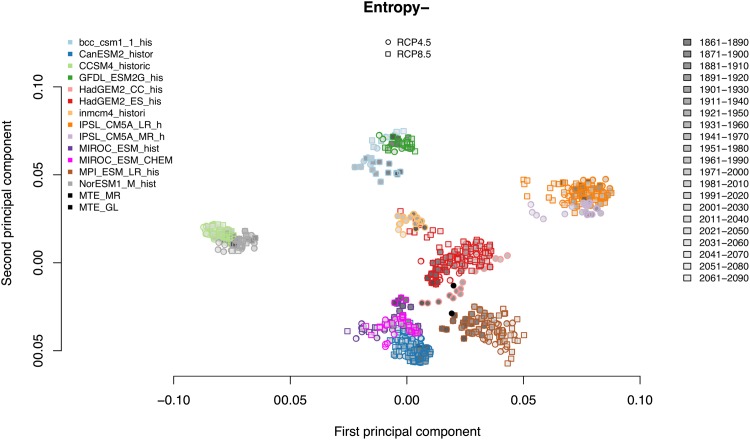
Differentiating model structure with statistical complexity measures. The different models are coded by different colours, filling indicates the analyzed time period for all grid cells of the globe.

The CMIP5 runs allow a detailed evaluation of the relative importance of the model choice and the scenario used (cf. Table 2 in S1 File). Global maps of the Weighted Permutation Entropy (S6 Fig) and the causal information planes for one of the 30-years periods and all 59 model runs are shown in S7 and S8 Figs. These plots show a large diversity of patterns and point densities in $H_{w}\times C_{w}$ across the runs. Some of the models extend over the whole entropy range, others are more constrained to higher entropy values. Although looking quite similar at first glance for most of the 59 model simulations, it is obvious that the local density of points is rather different between them. None of the simulations is very close to the upper limit curve, i.e. do not achieve the highest complexity possible, which would be the realm of deterministic dynamical systems. This might be explained by the fact that these models are not autonomous dynamical systems, but driven by stochastic drivers such as precipitation. In $H_{w}\times F_{w}$, many simulations exhibit a “fork” structure, or a separation of the values into (at least) two different “curves” with two different slopes (S8 Fig). The width of the point clouds around these “curves” is, however, rather different between the simulations. Global maps of the Weighted Permutation Entropy confirm that different models indeed show considerable differences in the simulated dynamics of GPP across various regions of the globe (S6 Fig). Using the rastering presented in the Methods section, we performed a PCA of the point clouds. The result for the first two dimensions is given by Fig 9. This figure shows that the entropy and Fisher information measures separate models according to model *structural* properties, and not scenarios, which is the core result of this section. Note that in all cases where clusters contain points of different colours, the colour-encoded models are actually variants of each other, for example, differing in spatial resolution or with or without a module for atmospheric chemistry. The classification is not sensitive to the two scenarios, which were identical for all models. The linear or bow-shaped structures of the points for some of the models indicate a systematic development during the simulation period.

## Conclusions

The main intention of the current paper was to test the usefulness of Information Theory Quantifiers as tools for data analysis and model benchmarking in the environmental sciences, and thus to reach a wider dissemination of these tools. Time series of simulated and observed GPP as a key ecosystem variable were investigated to show that the dynamics varies as a function of the underlying generating model or process, spatio-temporal scale and climate and ecosystem type, reflecting that this variable has an intricate temporal behaviour and is relevant for applications on local to global scales. Hence, statistical complexity measures can be used as model and data analytical tools that diagnose the short-term dynamics of the underlying time series. This is complementary to most conventional model diagnostics quantifying global statistical properties of the data, such as bias, correlation, variance, or higher statistical moments, which are in part insensitive to the temporal order (non-causal).

Three central results were presented in the present paper: First, ITQ derived from time series of remotely sensed vegetation activity proxies (FAPAR in our case) show both climate- and ecosystem-related continental-scale gradients and regional structures, and thus might serve as useful, efficient and robust data-analytical tools. Second, a model-data comparison based on ITQ reveals qualitatively different results compared to traditional model evaluation metrics; e.g., as demonstrated for European GPP simulations, a model with low absolute or relative error does not necessarily reproduce the dynamics of the observations well, and vice versa. A striking result is that the GPP data set that is constructed by the MTE approach has different, much more deterministic ITQ than the EC observations that enter the MTE procedure. Discrepancies between models and/or data provide useful hints to model structural deficiencies (as shown for Mediterranean FLUXNET sites). Third, the complexity of model simulated GPP is highly sensitive to model structure (i.e. differing between models), but largely insensitive to external climate forcing, atmospheric greenhouse gas concentrations or land use changes, as demonstrated for global- and biome-scale GPP simulations from the CMIP5 archive. These results highlight the benefits of model benchmarking and evaluation against a variety of model evaluation metrics, including model structural diagnostics such as the ITQ presented here.

ITQ allow a ranking of models according to their ability to reproduce the observed dynamical behaviour within the time span given by the embedding dimension and the delay parameter. The crucial limiting factor for this time span is the length of the time series. In the future, the complexity indicators could serve as objective functions to improve model performance, e.g. in an iterative or machine-learning setting to find optimal parameter sets or model structures (Ilie et al., submitted to *Geoscientific Model Development*) or in a data assimilation system (see e.g. [83]). In general terms, the methods employed here analyze and compare data sets, not models. They are close to non-parametric—the choice of the embedding dimension is dictated by the length of the time series—and do not make any specific assumptions about properties of the data, statistical or otherwise. Our results indicate that for land surface models, it is likely not sufficient to change the parameters of the model to reproduce the observed behaviour; rather, the model structure has to be revised, since the same model structure produces similar patterns independent of e.g. their initialization and the details of the parametrization.

Finally, a broad range of future applications of Information Theory Quantifiers in environmental science is conceivable: These could consist of efficiently diagnosing satellite data streams of very high spatio-temporal resolution in an increasingly data-rich era, as well as to use statistical complexity of environmental variables for classification purposes. Therefore, we propose that these tools could be taken up more widely by the community for model evaluation and benchmarking activities.

## Supporting Information

S1 Fig Weighted statistical complexity for the JRC-TIP FPAR dataset. (PDF)

S2 Fig Weighted Fisher Information for the JRC-TIP FPAR dataset. (PDF)

S3 Fig High-resolution version of Fig 3 (bottom panel) in the main text. (PDF)

S4 Fig ITQ-based and RMSE-based model comparison of JSBACH and LPJmL vs. MTE. (left) Jensen-Shannon Divergence, and (right) RMSE.
(PDF)

S5 FigITQ’s for temperate and boreal FLUXNET sites.(top left) Boreal sites, $H_{w}\times C_{w}$, and (top right) boreal sites $H_{w}\times F_{w}$. (bottom left) Temperate sites, $H_{w}\times C_{w}$, and (bottom right) temperate sites, $H_{w}\times F_{w}$.(PDF)

S6 Fig Global maps of the Weighted Permutation Entropy of GPP fluxes across 59 CMIP5 models and simulations for all land grid cells. Plots are illustrative for the 30-yr period 1981-2010.
(PDF)

S7 Fig
$H_{w}\times C_{w}$ of GPP fluxes across 59 CMIP5 models and simulations for all land grid cells.Plots are illustrative for the 30-yr period 1981-2010.(JPG)

S8 Fig
$H_{w}\times F_{w}$ of GPP fluxes across 59 CMIP5 models and simulations for all land grid cells. Plots are illustrative for the 30-yr period 1981-2010.(JPG)

S1 File Supporting Information file that contains additional Text and Tables. (PDF)

S1 Code Supporting Code Tutorial that introduces the R-package on Statistical Complexity (“statcomp”) and allows to reproduce Figs 1 and 2. (R)
