# Supplementary figures and images for: Diagnosing the Dynamics of Observed and Simulated Ecosystem Gross Primary Productivity with Time Causal Information Theory Quantifiers
Source: PLoS One. 2016 Oct 20;11(10):e0164960. doi: 10.1371/journal.pone.0164960 (PMC5072746; doi:10.1371/journal.pone.0164960)

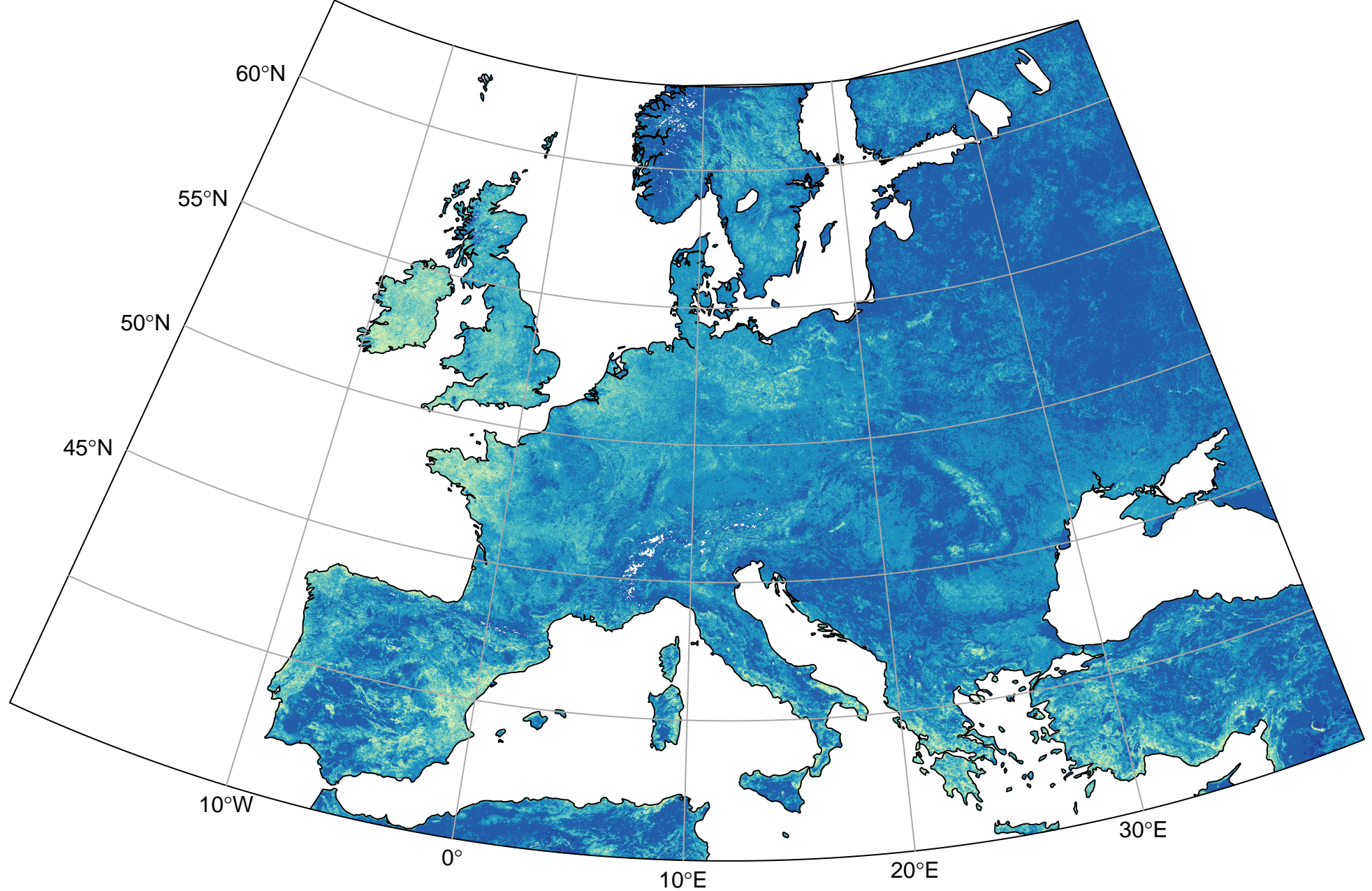

Weighted MPR Complexity

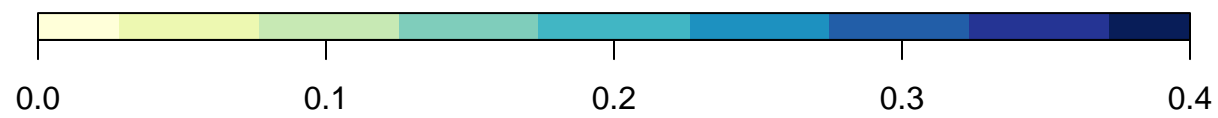

Supplement: S1 Fig — (PDF) [file pone.0164960.s001.pdf]

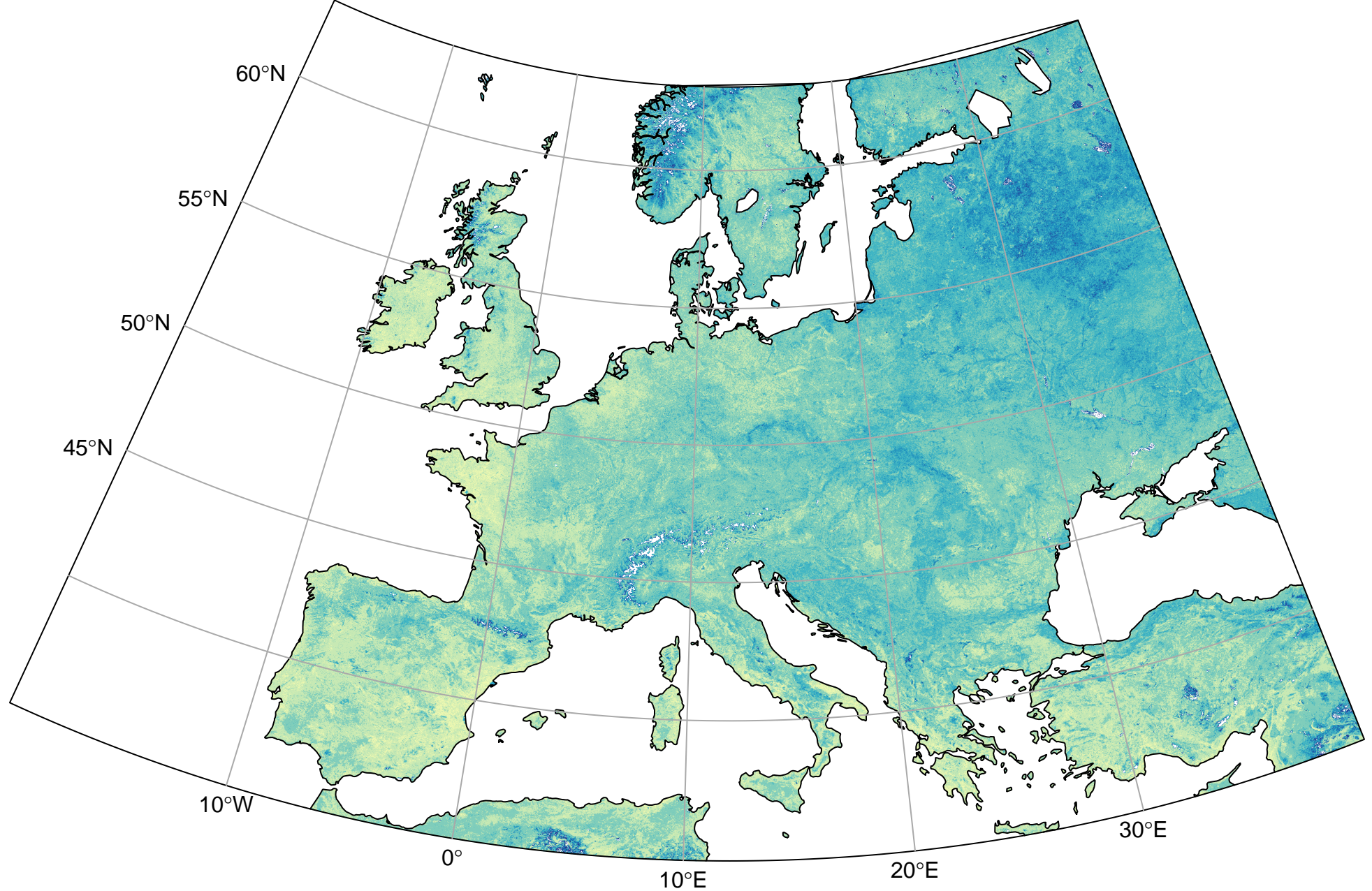

Weighted Fisher Information

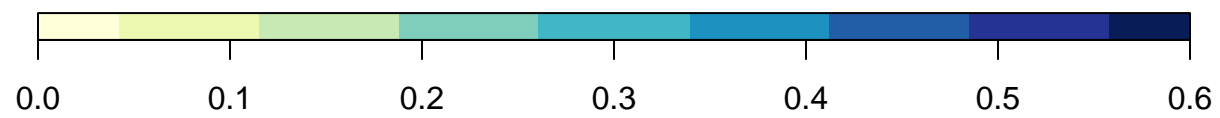

Supplement: S2 Fig — (PDF) [file pone.0164960.s002.pdf]

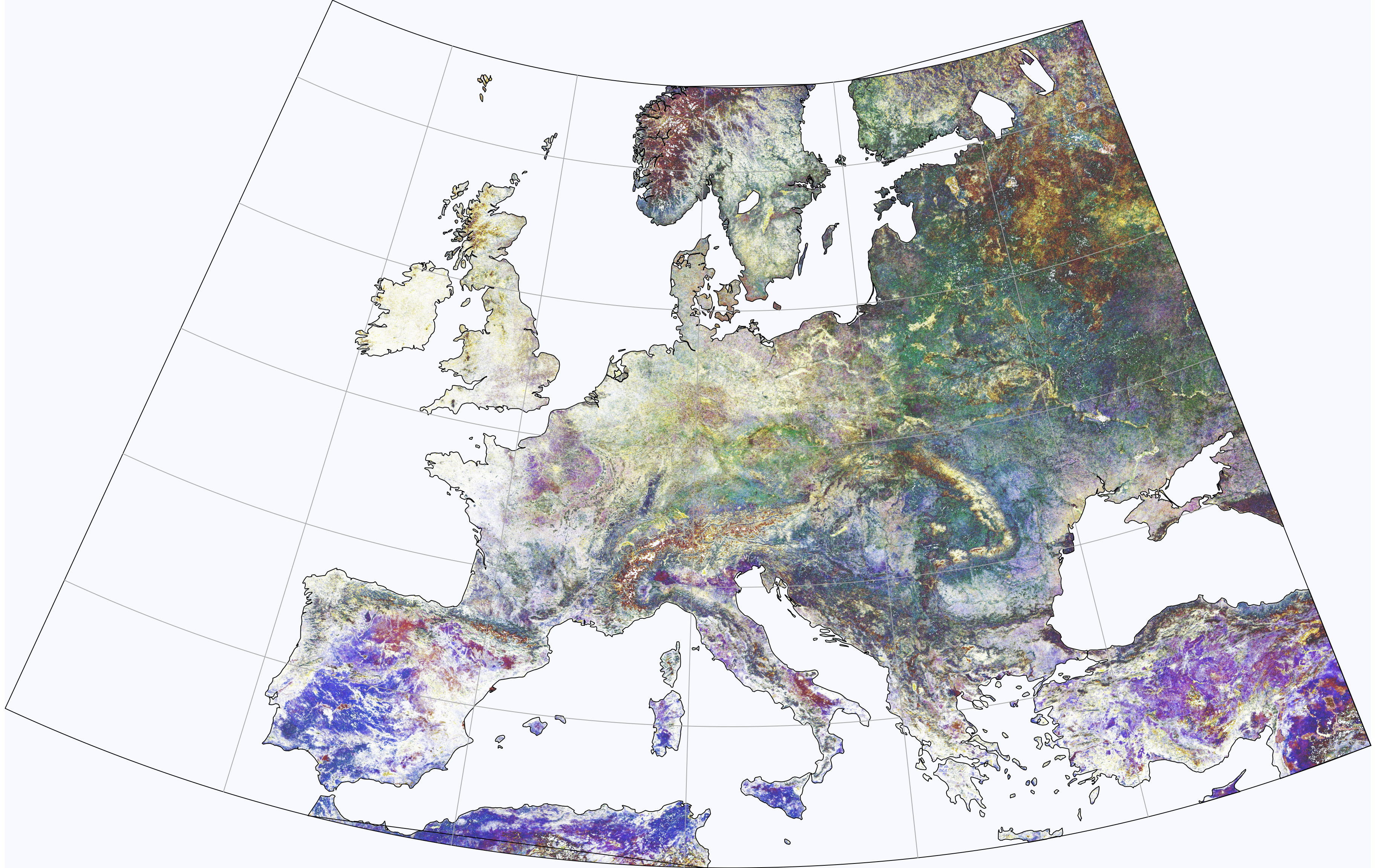

Supplement: S3 Fig — (PDF) [file pone.0164960.s003.pdf]

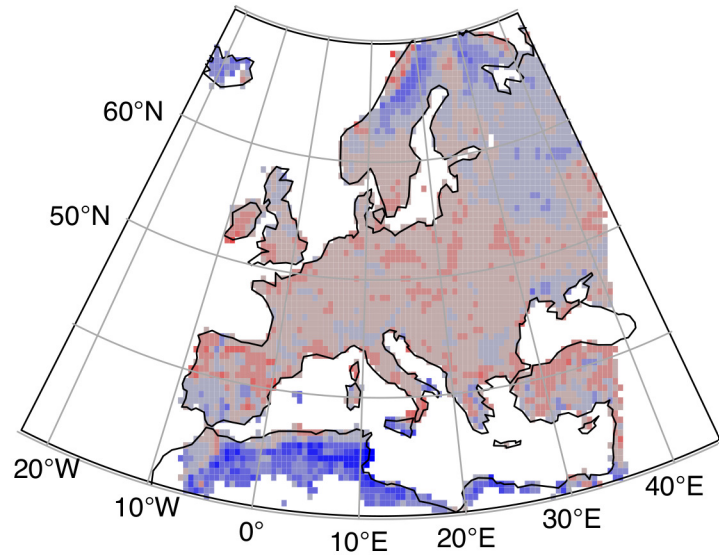

JS (JSBACH vs. MTE) – JS (LPJmL vs. MTE)

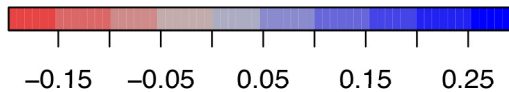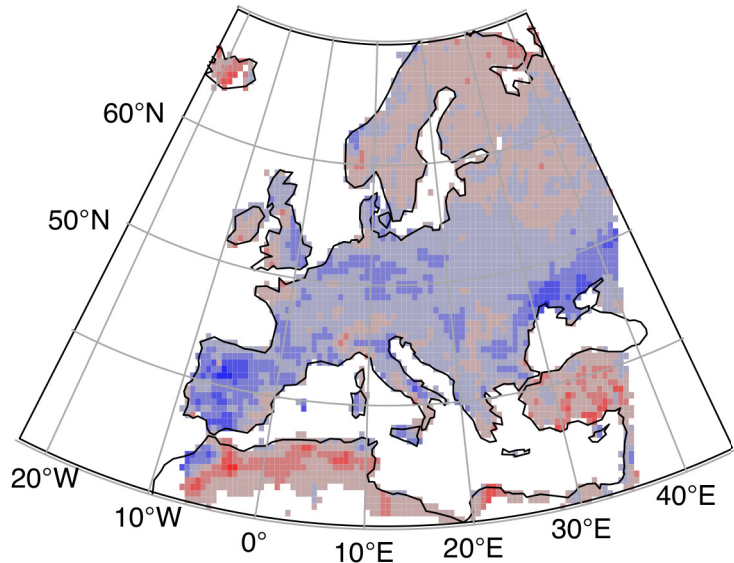

RMSE (JSBACH vs. MTE) – RMSE (LPJmL vs. MTE)

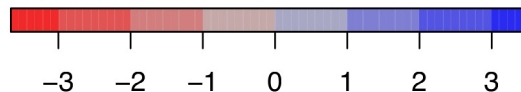

Supplement: S4 Fig — (left) Jensen-Shannon Divergence, and (right) RMSE. (PDF) [file pone.0164960.s004.pdf]

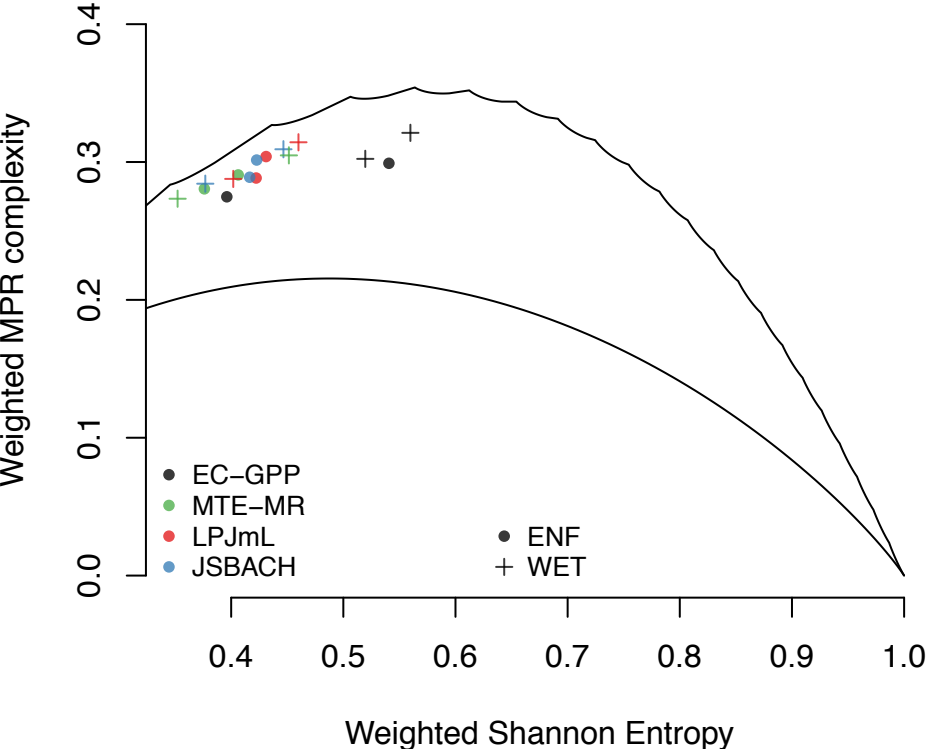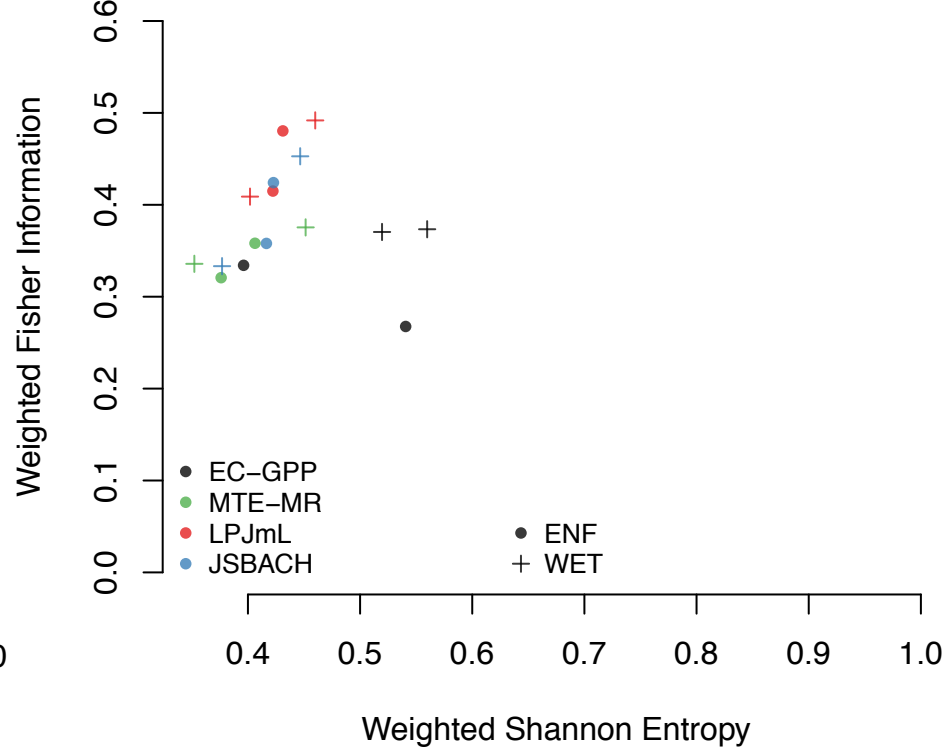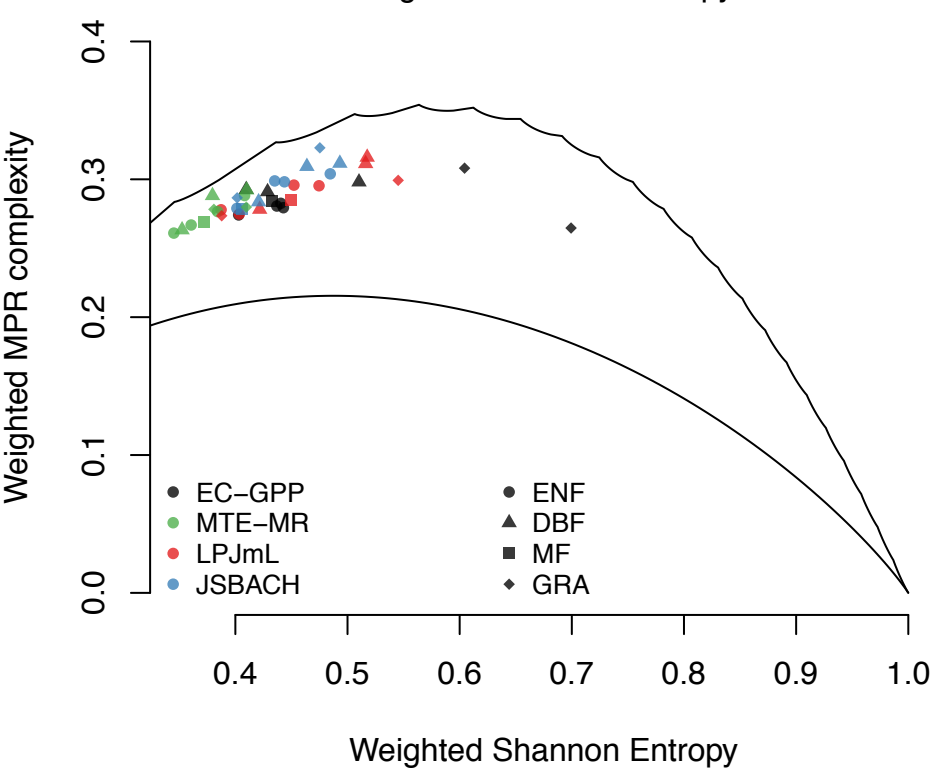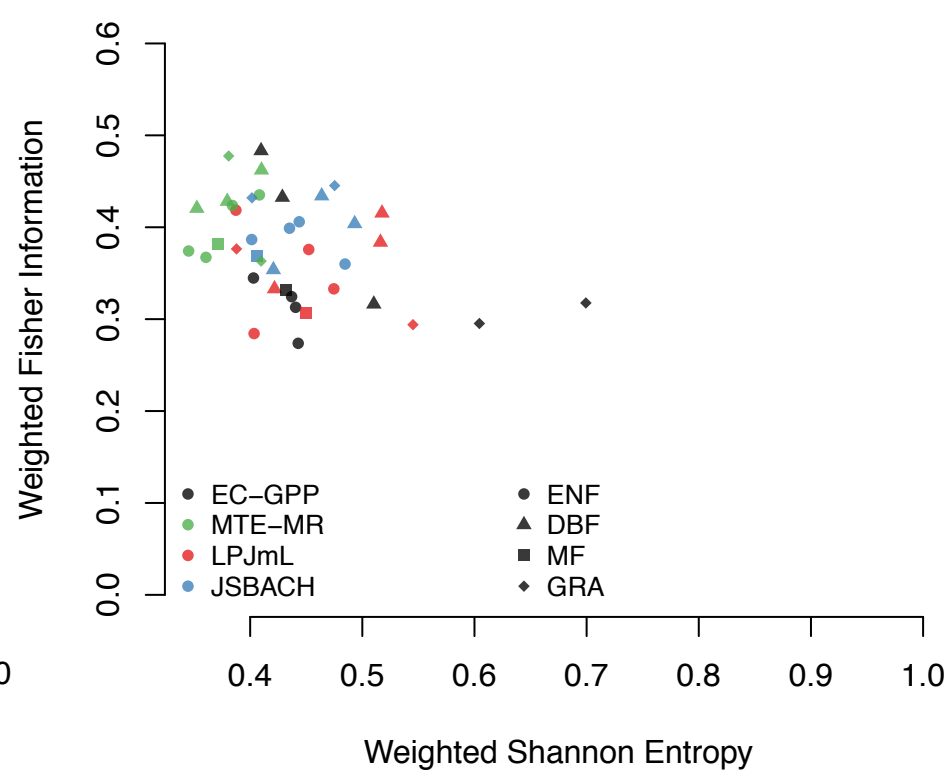

Supplement: S5 Fig — (top left) Boreal sites, Hw×Cw, and (top right) boreal sites Hw×Fw. (bottom left) Temperate sites, Hw×Cw, and (bottom right) temperate sites, Hw×Fw. (PDF) [file pone.0164960.s005.pdf]

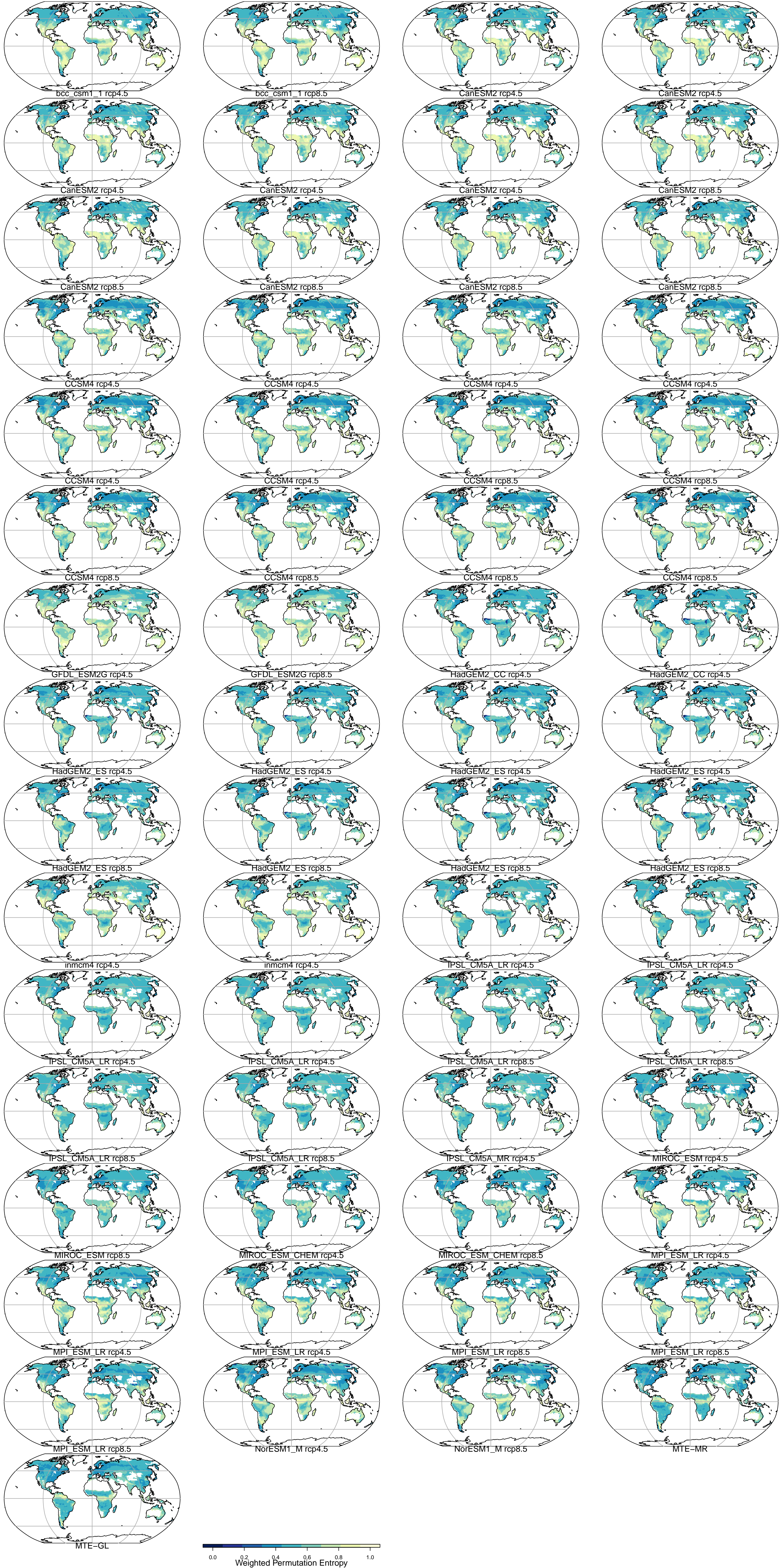

Supplement: S6 Fig — Plots are illustrative for the 30-yr period 1981-2010. (PDF) [file pone.0164960.s006.pdf]

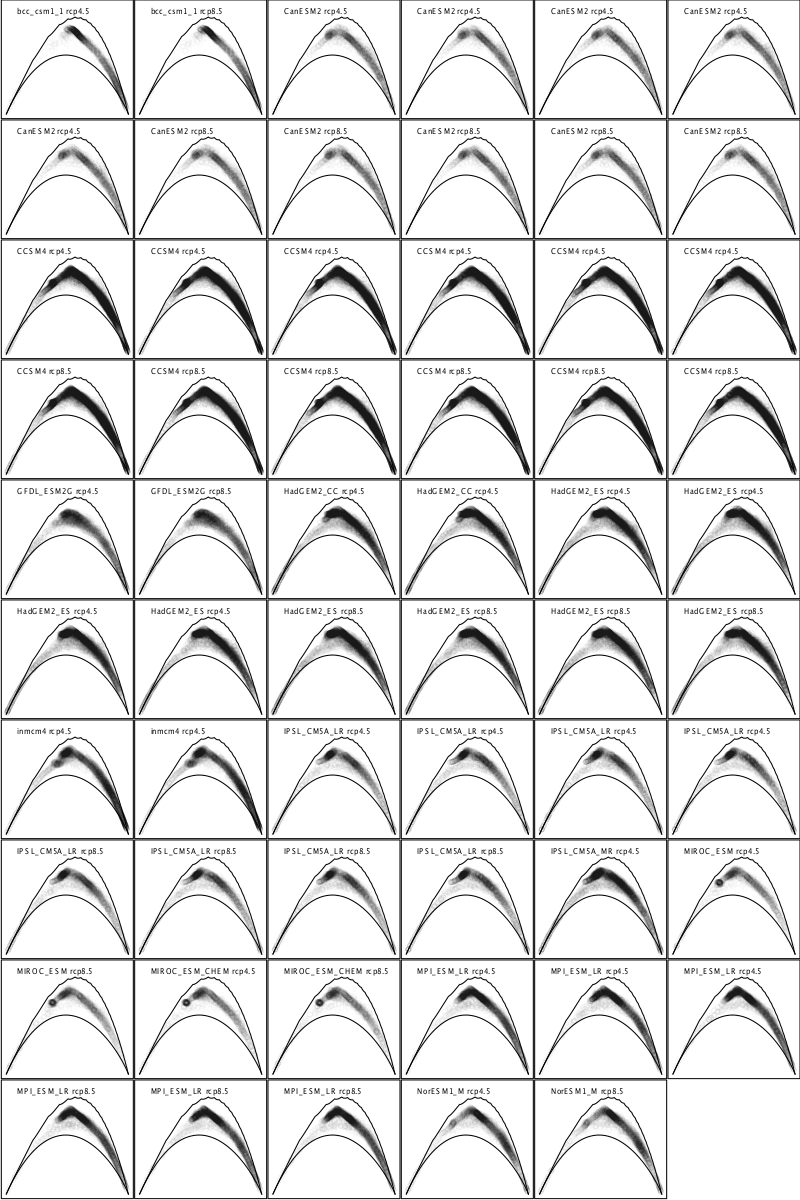

Supplement: S7 Fig — Plots are illustrative for the 30-yr period 1981-2010. (JPG) [file pone.0164960.s007.jpg]

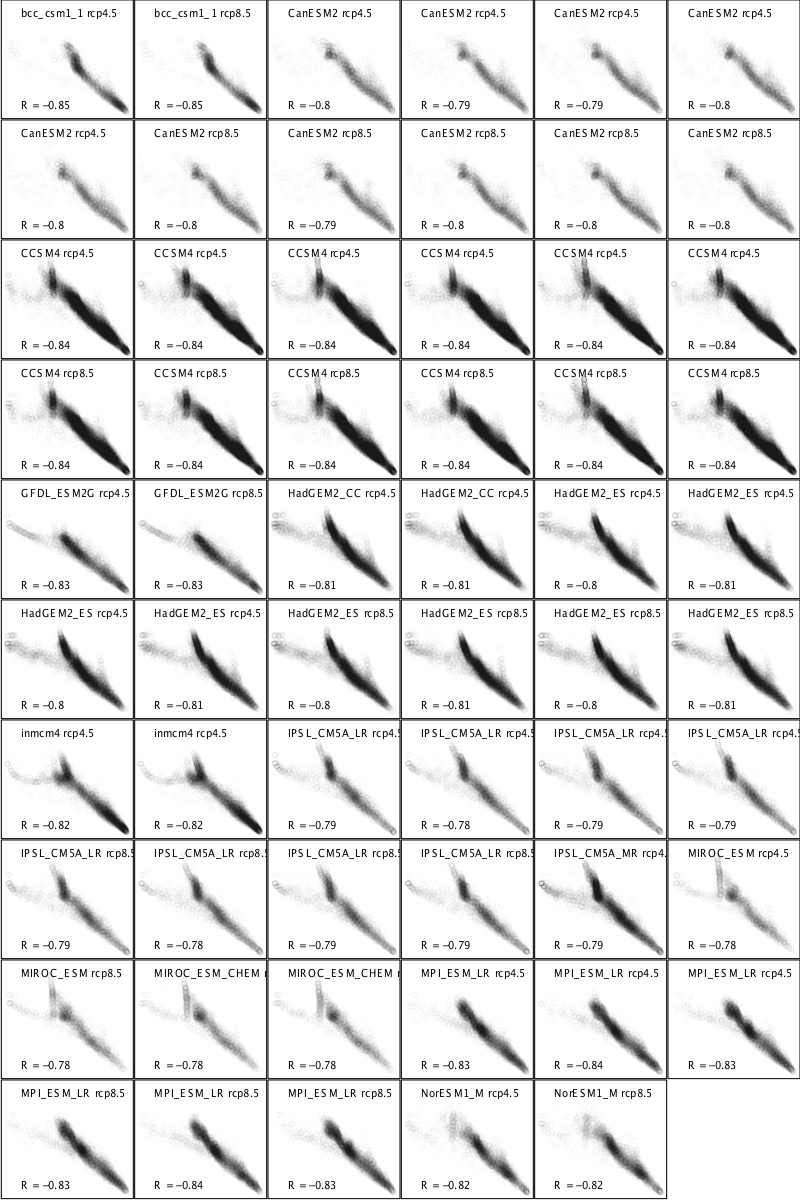

Supplement: S8 Fig — (JPG) [file pone.0164960.s008.jpg]
