# Supplementary material for: Diagnosing the Dynamics of Observed and Simulated Ecosystem Gross Primary Productivity with Time Causal Information Theory Quantifiers
Source: PLoS One. 2016 Oct 20;11(10):e0164960. doi: 10.1371/journal.pone.0164960 (PMC5072746; doi:10.1371/journal.pone.0164960)
Supplement: S1 File — (PDF) [file pone.0164960.s009.pdf]

S1 Supporting File to 'Diagnosing the Dynamics of Observed and Simulated Ecosystem Gross Primary Productivity with Time Causal Information Theory Quantifiers'

Sebastian Sippel[1*], Holger Lange[2,3], Miguel D. Mahecha[1,4,5], Michael Hauhs[6], Paul Bodesheim[1], Thomas Kaminski[7], Fabian Gans[1], Osvaldo A. Rosso[3,8,9]

1 Max Planck Institute for Biogeochemistry, Jena, Germany.

2 Norwegian Institute of Bioeconomy Research, Ås, Norway.

3 Instituto de Física, Universidade Federal de Alagoas, Maceió, Alagoas, Brazil.

4 German Centre for Integrative Biodiversity Research (iDiv), Leipzig, Germany.

5 Michael Stifel Center Jena for Data-Driven and Simulation Science, Jena, Germany.

6 University of Bayreuth, Bayreuth, Germany.

7 The Inversion Lab, Hamburg

8 Instituto Tecnológico de Buenos Aires (ITBA) and CONICET, Ciudad Autónoma de Buenos Aires, Argentina.

9 Complex Systems Group, Facultad de Ingeniería y Ciencias Aplicadas, Universidad de los Andes, Las Condes, Santiago, Chile.

* ssippel@bgc-jena.mpg.de

Contents

Tables **3**

Text **5**

Ordinal pattern statistics 5

Tables

Table 1. All FLUXNET sites used in this study.

Name	Years	Long.	Lat.	PFT	Climate Region	Ref.
BE-Vie	1996-2006	6.00	50.31	MF	Temperate	[2]
CH-Oe1	2000-2010	7.73	47.29	GRA	Temperate	[3]
CZ-Bk1	2000-2006	18.54	49.50	ENF	Temperate	[4]
DE-Hai	2000-2006	10.45	51.08	DBF	Temperate	[5]
DE-Tha	1996-2006	13.57	50.96	ENF	Temperate	[6]
DE-Wet	2002-2006	11.46	50.45	ENF	Temperate	[5]
DK-Sor	1996-2006	11.64	55.49	DBF	Temperate	[6]
ES-ES1	1999-2006	-0.32	39.35	ENF	Mediterranean	[4]
FI-Hyy	1996-2006	24.29	61.85	ENF	Boreal	[7]
FI-Kaa	2000-2006	27.30	69.14	WET	Boreal	[8]
FI-Sod	2000-2006	26.64	67.36	ENF	Boreal	[9]
FR-Hes	1997-2006	7.06	48.67	DBF	Temperate	[10]
FR-Pue	2000-2006	3.60	43.74	EBF	Mediterranean	[4]
HU-Bug	2002-2006	19.60	46.69	GRA	Temperate	[11]
IT-Amp	2002-2006	13.61	41.90	GRA	Mediterranean	[11]
IT-Col	1996-2006	13.59	41.85	DBF	Mediterranean	[4]
IT-Cpz	1997-2006	12.50	41.71	EBF	Mediterranean	[4]
IT-Ren	1999-2006	11.43	46.59	ENF	Temperate	[12]
IT-Ro1	2000-2006	11.93	42.41	DBF	Mediterranean	[13]
IT-Sro	1999-2006	10.28	43.73	ENF	Mediterranean	[14]
NL-Loo	1996-2006	5.74	52.17	ENF	Temperate	[15]
RU-Fyo	1998-2006	32.92	56.46	ENF	Temperate	a.
SE-Deg	2001-2005	19.55	64.18	WET	Boreal	b.

a. `http://www.fluxdata.org:8080/sitepages/siteInfo.aspx?RU-Fyo`
b. `http://www.fluxdata.org:8080/sitepages/siteInfo.aspx?SE-Deg`

Table 2. All CMIP5 models used in this study. Number of ensemble members includes both representative concentration pathways (RCP4.5 and RCP 8.5).

Modeling Center (or Group)	Inst. ID	Model Name	# Ens.
Beijing Climate Center, China Meteorological Administration	BCC	bcc-csm1-1	2
Canadian Centre for Climate Modelling and Analysis	CCCMA	CanESM2	10
National Center for Atmospheric Research	NCAR	CCSM4	12
NOAA Geophysical Fluid Dynamics Laboratory	NOAA GFDL	GFDL-ESM2G	2
Met Office	MOHC	HadGEM2-CC	2
Hadley Centre	MOHC	HadGEM2-ES	8
Institute for Numerical Mathematics	INM	inmcm4	2
Institut Pierre-	IPSL	IPSL-CM5A-LR	8
Simon Laplace	IPSL	IPSL-CM5A-MR	1
Japan Agency for Marine-Earth	MIROC	MIROC-ESM	2
Science and Technology, Atmosphere and Ocean Research Institute (The University of Tokyo), and National Institute for Environmental Studies	MIROC	MIROC-ESM-CHEM	2
Max Planck Institute for Meteorology	MPI-M	MPI-ESM-LR	6
Norwegian Climate Centre	NCC	NorESM1-M	2

Text

Ordinal pattern statistics

Given a one-dimensional time series $\mathcal{X}(t) = \{x_t; t = 1, \dots, M\}$ and a chosen window length D, "ordinal patterns" of order D are generated by first embedding the time series:

$$(s) \mapsto \left(x_{s-(D-1)\tau}, x_{s-(D-2)\tau}, \dots, x_{s-\tau}, x_s\right) \ , \tag{1}$$

which assigns to each time s the D-dimensional vector of values at times $s-(D-1)\tau, \dots, s-\tau, s$. Clearly, the greater D, the more information on the past is incorporated into the embedding vectors. By "ordinal pattern" related to the time (s), we mean the permutation $\pi = (r_0, r_1, \dots, r_{D-1})$ of $[0, 1, \dots, D-1]$ defined by

$$x_{s-r_{D-1}\tau} \le \ x_{s-r_{D-2}\tau} \le \dots \le \ x_{s-r_1\tau} \le x_{s-r_0\tau}. \tag{2}$$

In this way the vector defined by Eq. (1) is converted into a unique symbol π. We set $r_i < r_{i-1}$ if $x_{s-r_i} = x_{s-r_{i-1}}$ for uniqueness.

In order to illustrate the Bandt-Pompe (BP) method, we will consider a simple example: a time series with seven ($M = 7$) values $\mathcal{X} = \{4, 7, 9, 10, 6, 11, 3\}$ only, and we evaluate the BP-PDF for $D = 3$ and $\tau = 1$. In this case the state space is divided into 3! partitions and 6 mutually exclusive permutation symbols are considered. The triplet $(4, 7, 9)$ and $(7, 9, 10)$ represent the permutation pattern [012] since they are in increasing order. On the other hand, $(9, 10, 6)$ and $(6, 11, 3)$ correspond to the permutation pattern [201] since $x_{s+2} < x_s < x_{s+1}$, while $(10, 6, 11)$ has the permutation pattern $\{102\}$ with $x_{s+1} < x_s < x_{s+2}$. Then, the associated probabilities to the 6 patterns are: $p([012]) = p([201]) = 2/5$; $p([102]) = 1/5$; $p([021]) = p([120]) = p([210]) = 0$.

For all the $D!$ possible orderings (permutations) π_i, their relative frequencies can be naturally computed according to the number of times this particular order sequence is found in the time series, divided by the total number of sequences,

$$p(\pi_i) = \frac{\#\{s | s \le N - (D-1)\tau; (s) \text{ is of type } \pi_i\}}{N - (D-1)\tau}, \tag{3}$$

where # denotes cardinality. Thus, an ordinal pattern probability distribution $P = \{p(\pi_i), i = 1, \dots, D!\}$ is obtained from the time series.

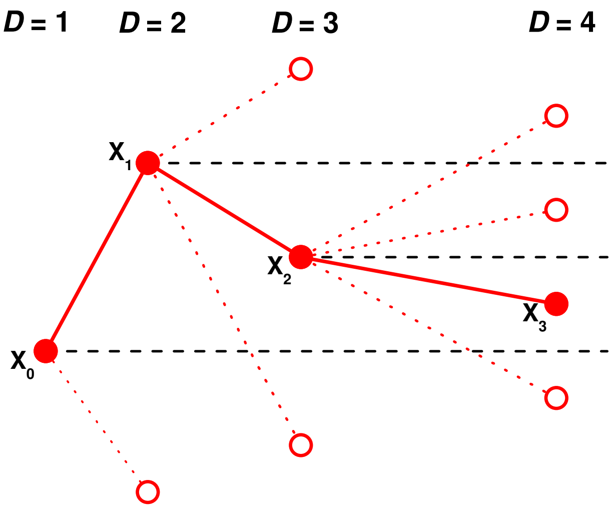

Fig 1. Illustration of the construction principle for ordinal patterns of length D [1]. If $D = 4$ and $\tau = 1$, full circles and continuous lines represent the sequence of values $x_0 < x_1 > x_2 > x_3$ which lead to the pattern $\pi = [0321]$.

Figure 1 illustrates the construction principle of the ordinal patterns of length $D = 2$, 3 and 4 with $\tau = 1$ [1]. Consider the sequence of observations $\{x_0, x_1, x_2, x_3\}$. For $D = 2$, there are only two possible directions from x_0 to x_1: up and down. For $D = 3$, starting from x_1 (up) the third part of the pattern can be above x_1, below x_0, or between x_0 and x_1. A similar situation can be found starting from x_1 (down). For $D = 4$, for each one of the six possible positions for x_2, there are four possible localizations for x_3, yielding $D! = 4! = 24$ different possible ordinal patterns. In Fig. 1, full circles and continuous lines represent the sequence values $x_0 < x_1 > x_2 > x_3$, which leads to the pattern $\pi = [0321]$. A graphical representation of all possible patterns corresponding to $D = 3, 4$ and 5 can be found in Fig. 2 of Parlitz *et al.* [1].

The embedding dimension D plays an important role in the evaluation of the appropriate probability distribution, because D determines the number of accessible states $D!$ and also conditions the minimum acceptable length $M \gg D!$ of the time series that one needs in order to work with reliable statistics [16].

References

1. Parlitz U, Berg S, Luther S, Schirdewan A, Kurths J, Wessel N. Classifying cardiac biosignals using ordinal pattern statistics and symbolic dynamics. Computers in biology and medicine. 2012;42(3):319–327.

2. Aubinet M, Chermanne B, Vandenhaute M, Longdoz B, Yernaux M, Laitat E. Long term carbon dioxide exchange above a mixed forest in the Belgian Ardennes. Agricultural and Forest Meteorology. 2001;108(4):293–315.

3. Ammann C, Flechard C, Leifeld J, Neftel A, Fuhrer J. The carbon budget of newly established temperate grassland depends on management intensity. Agriculture, Ecosystems & Environment. 2007;121(1):5–20.

4. Reichstein M, Falge E, Baldocchi D, Papale D, Aubinet M, Berbigier P, et al. On the separation of net ecosystem exchange into assimilation and ecosystem respiration: review and improved algorithm. Global Change Biology. 2005;11(9):1424–1439.

5. Anthoni P, Knohl A, Rebmann C, Freibauer A, Mund M, Ziegler W, et al. Forest and agricultural land-use-dependent CO2 exchange in Thuringia, Germany. Global Change Biology. 2004;10(12):2005–2019.

6. Grünwald T, Bernhofer C. A decade of carbon, water and energy flux measurements of an old spruce forest at the Anchor Station Tharandt. Tellus B. 2007;59(3):387–396.

7. Suni T, Rinne J, Reissell A, Altimir N, Keronen P, Rannik U, et al. Long-term measurements of surface fluxes above a Scots pine forest in Hyytiala, southern Finland, 1996-2001. Boreal Environment Research. 2003;8(4):287–302.

8. Aurela M, Laurila T, Tuovinen JP. Seasonal CO2 balances of a subarctic mire. Journal of Geophysical Research: Atmospheres. 2001;106(D2):1623–1637.

9. Suni T, Berninger F, Vesala T, Markkanen T, Hari P, Mäkelä A, et al. Air temperature triggers the recovery of evergreen boreal forest photosynthesis in spring. Global Change Biology. 2003;9(10):1410–1426.

10. Granier A, Ceschia E, Damesin C, Dufrêne E, Epron D, Gross P, et al. The carbon balance of a young beech forest. Functional ecology. 2000;14(3):312–325.

11. Gilmanov T, Soussana J, Aires L, Allard V, Ammann C, Balzarolo M, et al. Partitioning European grassland net ecosystem CO 2 exchange into gross primary productivity and ecosystem respiration using light response function analysis. Agriculture, ecosystems & environment. 2007;121(1):93–120.

12. Cescatti A, Marcolla B. Drag coefficient and turbulence intensity in conifer canopies. Agricultural and forest meteorology. 2004;121(3):197–206.

13. Arain MA, Restrepo-Coupe N. Net ecosystem production in a temperate pine plantation in southeastern Canada. Agricultural and Forest Meteorology. 2005;128(3):223–241.

14. Chiesi M, Maselli F, Bindi M, Fibbi L, Cherubini P, Arlotta E, et al. Modelling carbon budget of Mediterranean forests using ground and remote sensing measurements. Agricultural and Forest Meteorology. 2005;135(1):22–34.

15. Dolman A, Moors E, Elbers J. The carbon uptake of a mid latitude pine forest growing on sandy soil. Agricultural and Forest Meteorology. 2002;111(3):157–170.

16. Kowalski A, Martín M, Plastino A, Rosso O. Bandt–Pompe approach to the classical-quantum transition. Physica D: Nonlinear Phenomena. 2007;233(1):21–31.
